# Exploring *FGFR3* Mutations in the Male Germline: Implications for Clonal Germline Expansions and Paternal Age-Related Dysplasias

**DOI:** 10.1093/gbe/evae015

**Published:** 2024-02-27

**Authors:** Sofia Moura, Ingrid Hartl, Veronika Brumovska, Peter P Calabrese, Atena Yasari, Yasmin Striedner, Marina Bishara, Theresa Mair, Thomas Ebner, Gerhard J Schütz, Eva Sevcsik, Irene Tiemann-Boege

**Affiliations:** Institute of Biophysics, Johannes Kepler University, Linz, Austria; John P. Hussman Institute for Human Genomics, Miller School of Medicine, University of Miami, Miami, USA; Institute of Biophysics, Johannes Kepler University, Linz, Austria; Institute of Applied Physics, TU Wien, Vienna, Austria; Quantitative and Computational Biology, University of Southern California, Los Angeles, USA; Institute of Biophysics, Johannes Kepler University, Linz, Austria; Institute of Biophysics, Johannes Kepler University, Linz, Austria; Institute of Applied Physics, TU Wien, Vienna, Austria; Institute of Biophysics, Johannes Kepler University, Linz, Austria; Department of Gynecology, Obstetrics and Gynecological Endocrinology, Johannes Kepler University, 4020 Linz, Austria; Institute of Applied Physics, TU Wien, Vienna, Austria; Institute of Applied Physics, TU Wien, Vienna, Austria; Institute of Biophysics, Johannes Kepler University, Linz, Austria

**Keywords:** FGFR3, mutations, selective advantage, clonal expansion, driver mutations, de novo mutations, ligand-independent activation, digital PCR

## Abstract

Delayed fatherhood results in a higher risk of inheriting a new germline mutation that might result in a congenital disorder in the offspring. In particular, some *FGFR3* mutations increase in frequency with age, but there are still a large number of uncharacterized *FGFR3* mutations that could be expanding in the male germline with potentially early- or late-onset effects in the offspring. Here, we used digital polymerase chain reaction to assess the frequency and spatial distribution of 10 different *FGFR3* missense substitutions in the sexually mature male germline. Our functional assessment of the receptor signaling of the variants with biophysical methods showed that 9 of these variants resulted in a higher activation of the receptor´s downstream signaling, resulting in 2 different expansion behaviors. Variants that form larger subclonal expansions in a dissected postmortem testis also showed a positive correlation of the substitution frequency with the sperm donor's age, and a high and ligand-independent FGFR3 activation. In contrast, variants that measured high FGFR3 signaling and elevated substitution frequencies independent of the donor's age did not result in measurable subclonal expansions in the testis. This suggests that promiscuous signal activation might also result in an accumulation of mutations before the sexual maturation of the male gonad with clones staying relatively constant in size throughout time. Collectively, these results provide novel insights into our understanding of the mutagenesis of driver mutations and their resulting mosaicism in the male germline with important consequences for the transmission and recurrence of associated disorders.

**Significance**

*FGFR3* is a well-known oncogene that is highly expressed in the male gonad. Here, we investigated how a series of mutations in *FGFR3*, associated with different dysplasias, expand in the male germline. Specifically, we investigated the functional activation, as well as the occurrence in human testis and sperm of 10 different *FGFR3* variants categorized as deleterious, benign, or not reported. We found that FGFR3 promiscuous (ligand-independent) activation leads to the accumulation of mutations in the male germline in 2 ways: mutations that increase in frequency with age in testis and sperm, and those mutations that arise before the male germline becomes sexually mature and stay constant in numbers with age, a novel finding for this type of mutagenesis.

## Introduction

Driver or selfish mutations are described in tumors (Yates et al. 2015; Jamal-Hanjani et al. 2017; Turajlic et al. 2018). In the male germline, they have been observed in genes involved in the RAS-MAKP signaling pathway (e.g. *FGFR2, FGFR3, RET, HRAS, PTPN11, KRAS BRAF, CBL, MAP2K1, MAP2K2, RAF1*, and *SOS1*) (Tiemann-Boege et al. 2002; Qin et al. 2007; Goriely et al. 2009; Choi et al. 2012; Shinde et al. 2013; Maher et al. 2018). These mutations reach variant allele frequencies (VAFs) of ∼10^−4^ in the male germline, orders of magnitude higher than the estimated average human genome mutation rate per cell division per generation at any given genomic position (∼1.1 × 10^−8^) (Kong et al. 2012; Francioli et al. 2015). Further, these mutations are missense and gain-of-function that are enriched in sperm of older donors and form measurable subclonal expansions or clusters in the testis of older men. They occur exclusively in the male germline and older normal men have a higher probability of having an affected child with an autosomal dominant genetic disorder caused by a single-point mutation than younger males (known as paternal-age effect or PAE) (Arnheim and Calabrese 2009; Goriely and Wilkie 2012).

The production of sperm cells requires the proliferation and differentiation of spermatogonial stem cells. There are 3 types of spermatogonial stem cells in the sexually mature male gonad: A_dark_ and A_pale_, both considered undifferentiated spermatogonia, and B-type spermatogonia that differentiate and enter meiosis to produce sperm cells. The A_pale_ cells (SrAp) are active stem cells, undergoing self-renewing and differentiating divisions. The type of division is likely random with half resulting in sperm and the other half being self-renewing producing 2 stem cells. In order to maintain fertility, this balance between self-renewing and differentiated cells is essential to produce sperm (reviewed in Arnheim and Calabrese 2016; Eboreime et al. 2022).

These ongoing cell divisions occurring throughout the lifetime of the sexually mature male germline might explain the accumulation of driver mutations. In particular, driver mutations in genes of receptor tyrosine kinases (RTKs), such as *FGFR3* highly expressed in spermatogonial stem cells, modify the signaling of the receptor by promoting ligand-independent activation (Bellus et al. 2000; Krejci et al. 2008; He et al. 2012; Sarabipour and Hristova 2016). This promiscuous receptor might promote a positive selective advantage compared to the neighboring cells either by modifying the patterns of cell division, cell growth, or cell death (Arnheim and Calabrese 2009; Choi et al. 2012; Yoon et al. 2013).

As a result, mutations with a selective advantage increase in numbers and form subclonal expansions in the aging testis (Qin et al. 2007; Choi et al. 2012; Shinde et al. 2013; Maher et al. 2018). Critical for the formation of these clusters or mutation foci is that all of the descendants stay in close proximity to their SrAp source. Interestingly, clusters or subclonal populations have been described in different anatomical locations in the same testis for multiple different substitutions (Arnheim and Calabrese 2016). Driver mutations also result in a higher number of mutant sperm in older donors, as described in *FGFR3* for p.G380R (Tiemann-Boege et al. 2002); and for different mutations in codon 650 (Goriely et al. 2009) and p.R669G (Maher et al. 2018).

Despite the importance of driver mutations in the male germline due to their high incidence and the increased frequency, as well as their potential early- or late-onset effects, our knowledge about this mutagenic mechanism is still limited. For example, are these mutations primarily expanding within the sexually mature germline, leading to an increased mutational load in the sperm of older donors and a higher incidence of mutations in the population as paternal age advances (if mutations are viable)? Alternatively, could driver mutations already be enriched in the sperm of younger donors through prepubertal expansions, resulting in mosaicism within the male germline? It is worth noting that if these mutations occur in cells that do not remain in close proximity, the subclonal expansion might not be observable in the testis.

Further, with the explosion of sequencing data of somatic tissues in clinical settings (e.g. COSMIC), a large number of mutations are being reported for *FGFR3*. Many might have phenotypes potentially leading to early- or late-onset disorders in children of older men. However, the impact of genetic changes on the activation state and the resulting enrichment in the male germline of different *FGFR3* variants is not well understood. There is only one analysis of this kind for codon 650, an important tyrosine kinase (TK) activation site (Goriely et al. 2009). In this work, it was shown that strongly activating mutations often associated with tumors (p.K650M) were found at lower frequencies in sperm than milder activating mutations.

As such, identifying prospective driver mutations accumulating in the male germline has been an intensive target of research, but a tricky task given the ultralow frequency of these events (Maher et al. 2018; Salazar et al. 2022). Advances in next-generation sequencing technologies have enabled the identification of mutations with high resolution. In particular, identifying prospective driver mutation in the testes (Arnheim and Calabrese 2016; Maher et al. 2018; Eboreime et al. 2022) or sperm (Salazar et al. 2022) using ultradeep sequencing methods have identified a series of mutations in the RTK-RAS pathway expanding in the male germline. Especially, duplex sequencing (DS) has the sensitivity to capture mutations occurring as low as 10^−7^ (Schmitt et al. 2012; Kennedy et al. 2014) and has identified rare variants in *FGFR3* of sperm DNA occurring at higher than expected frequencies (Salazar et al. 2022). However, despite the promising sensitivity of DS, in practice, it still lacks the throughput to screen enough genomes needed to assess larger sample sizes.

To gain further insights into the accumulation of driver mutations in the male germline and their effect on their signaling properties, we assessed the substitution frequencies of 10 variants with digital polymerase chain reaction (dPCR) in a total of ∼50 to 100 sperm donors per site. Further, we assessed the expansion behavior of 5 variants within a postmortem testis of a 68− or 73-year-old testis dissected into 48 pieces. In addition, we evaluated the activation of the mutant protein using total internal reflection fluorescence microscopy. The 10 *FGFR3* missense substitutions were selected based on reports of higher de novo mutation (*DNM*) frequencies in sperm of 10^−5^ to 10^−4^ measured by DS (Salazar et al. 2022). Further selection criteria of these 10 variants was their association with phenotypes, clinical significance (pathogenic, likely benign, uncertain), predicted deleteriousness (CADD score) (Kircher et al. 2014), and SIFT score (Ng and Henikoff 2003; Sim et al. 2012), gnomAD and/or COSMIC reports. Five of the variants have been associated with other growth disorders; 3 of them were predicted in silico to have a strong deleterious effect on the protein structure (CADD) and described as deleterious (ClinVar), and 2 had uncertain or likely benign effects. These 10 variants also included 2 well-characterized PAE substitutions: c.1138G > A (p.G380R) and c.1118A > G (p.Y373C).

## Results

### Mutations in Sperm DNA

**Fig. 1.**
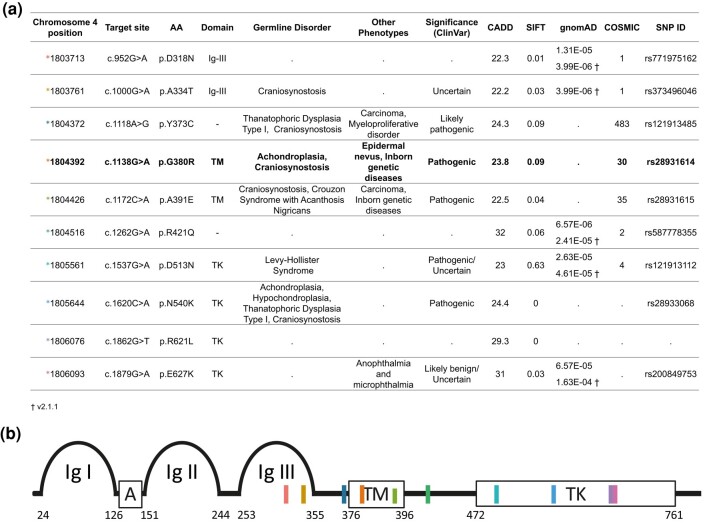
a) Target *FGFR3* variants and associated information. The canonical PAE variant c.1138 is shown in bold. Variants are organized by numerical order according to the coding sequence position. Base positions on chromosome 4 are based on GRCh38.p13. Phenotype data were retrieved from ClinVar. CADD (Combined Annotation Dependent Depletion; Kircher et al. 2014) score is defined according to GRCh38-v1.6 and annotates the level of deleteriousness. SIFT (Sorting Intolerant from Tolerant) algorithm was used to predict the effect of the amino acid substitutions on the FGFR3 protein-based conservation. SIFT values lower than 0.05 are considered deleterious, whereas values above are considered to be tolerated. All gnomAD information was retrieved from v3.1.2 except those indicated (†) (Karczewski et al. 2020). COSMIC (Catalogue of Somatic Mutations in Cancer) data were based on version 94 (Tate et al. 2019). All data were called based on transcript ENST00000440486, *FGFR3* isoform IIIc, and was last updated on 2022 November 14. b) Variants are indicated at their approximate location in the protein domains, color-coded with the asterisk in the table. Ig-like domains (Ig-I, Ig-III); acidic box (A), transmembrane domain (TM), and an intracellular split tyrosine kinase domain (TK1 and TK2). Numbers below the schematic indicate the amino acid starting and end positions of the domains within the protein.

Here, we investigated the VAF in a total of ∼50 to 100 sperm donors per site (aged from 23 to 59 years old) for 10 different *FGFR3* variants (Fig. 1). Variant c.1138G > A (p.G380R) is considered a canonical PAE mutation, and causes 98% of the spontaneous achondroplasia (ACH) cases (MIM #100800) (Shiang et al. 1994; Bellus et al. 1995). The mutation associated with ACH affects the transmembrane domain of FGFR3 and has been described to result in a higher signaling activity (Naski et al. 1996; Webster et al. 1996; Hartl et al. 2023). In particular, c.1138G > A was also shown to (i) increase in frequency in sperm of older donors (Tiemann-Boege et al. 2002), (ii) to form subclonal clusters in the testis (Shinde et al. 2013; Maher et al. 2018), and (iii) to occur at a higher incidence in offspring of older fathers (Risch et al. 1987). Variant, c.1118G > A (p.Y373C), is one of the most predominant causal mutations of Thanatophoric Dysplasia Type I (TDI) also reported to increase in incidence with paternal age (Rousseau et al. 1996). Seven variants were also measured in sperm DNA with DS (Salazar et al. 2022). Five variants were classified in ClinVar as deleterious/pathogenic, 2 were benign or uncertain, yet had a very high CADD score, and the remaining variants had an unknown ClinVar categorization, but had a high CADD score and were reported in gnomAD and/or COSMIC (Fig. 1).

### Measuring Low-Frequency Variants

**Fig. 2.**
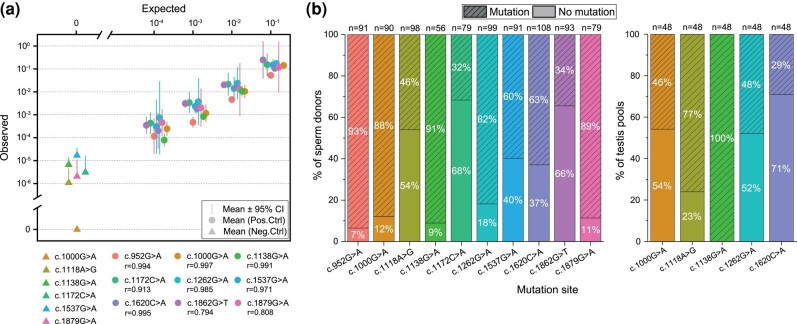
a) Spike-in serial dilutions for the analyzed variants and their respective Pearson's correlation coefficient (*r*). We measured 3 technical replicates per dilution step with a mixture of WT (human genomic DNA) and variant templates (Coriell Cell line or variant plasmids) according to each dilution step (1:10 to 1:10,000, 10-fold steps). Dilutions contained either ∼20,000, ∼36,000, or ∼72,000 genomes of WT (human genomic DNA) and variant templates distributed in 1, 2, or 4 reactions, respectively, such that there was on average 1 to 2 DNA templates per droplet. For the canonical variant (c.1138G > A), we used the data reported in Striedner et al. (2024). Negative controls (triangles) are plotted on the left side of the *x* axis (data in supplementary table S2, Supplementary Material online). b) Percentage of sperm samples or testis samples with or without mutations for the 10 *FGFR3* loci. The number of total donors screened for each locus is represented (*n*) at the top of each bar column. Percentages are rounded with no decimal number.

We used dPCR as a high-throughput method to measure rare VAFs that can process many samples. Specifically, we used the commercial platform (BioRad) known as droplet digital PCR (ddPCR) and an in-house method, referred to as bead emulsion amplification (BEA) validated previously (Boulanger et al. 2012; Shinde et al. 2013; Palzenberger et al. 2017). First, we determined the accuracy and reproducibility of the dPCR. For this purpose, we performed serial dilution experiments with positive controls (cell line encoding c.1620C > A, or sequence-confirmed variant plasmids for the remaining target loci) spiked into wild-type (WT) human genomic DNA at different ratios (Fig. 2a; supplementary fig. S1 and table S1, Supplementary Material online). We observed a good correspondence between the input ratio of mutant to WT copies with respect to the observed ratio (Pearson's correlation coefficient, *r*, ranging from 0.79 to 0.99).

Data from these dilutions provided an estimate of false positives or false negatives, in which case VAFs would be higher or lower than expected, respectively. Further, these data served to calibrate the amounts of genomes per droplet and to set the thresholds of the ddPCR analysis software (BioRad) to call a “positive”. This threshold was used for all the data for a particular variant. This manual thresholding treated the data uniformly (also outliers), potentially introducing a bias in the absolute VAF estimates. However, while different thresholds can impact absolute numbers, VAF differences among samples remain unchanged in small threshold adjustments. Note that for the in-house dPCR method (BEA), we used a cluster analysis algorithm to set these thresholds, as described in detail in Striedner et al. (2024) and also in previous studies validating this method (see Boulanger et al. 2012; Shinde et al. 2013).

We also assessed the VAFs in samples assumed to be mutation-free. These samples were sequence-confirmed plasmids. The WT plasmid measured a median VAF = 0 to ∼10^−6^, except for variant c.1537G > A (Fig. 2a and supplementary table S2, Supplementary Material online). Note that plasmids used a different DNA extraction protocol than genomic DNA from sperm and testis samples and could have been subject to DNA lesions representing false positives. For a more detailed discussion on these samples, please refer to Supplementary material online.

In this work, we screened 270,000 to 300,000 molecules per sample (distributed in 4 reactions). For VAFs ∼10^−5^, we observed 1 to 2 mutations per sample. For some samples, we also observed 0 mutations (negative samples); see Fig. 2b and supplementary table S3, Supplementary Material online. In screening ∼300,000 molecules, a mutation at frequency of 10^−5^ is expected to not appear in the sample ∼60% of the time (based on the Poisson distribution of rare events). Therefore, failure to observe positive counts at this depth can still be consistent with mutations present at even lower VAFs. The fraction of mutation-free samples ranged from 7% to 66% (Fig. 2b; supplementary table S3, Supplementary Material online). Note that we observed a higher fraction of mutation-free samples in the testis than in sperm samples for the same variant. These differences in negatives for the same site suggest that the assays are sensitive at levels larger than 10^−5^, but lack the accuracy to measure lower VAFs. In order to measure lower frequencies in a sample (e.g. ∼10^−6^), at least a million molecules distributed into 16 to 20 reactions must be screened.

As a further validation of the accuracy of the dPCR, we compared the VAFs measured with dPCR (supplementary table S4, Supplementary Material online) to those obtained using DS extracted from Salazar et al. (2022) for the same sperm donors or age-matched samples (supplementary table S5, Supplementary Material online). The latter method captures information from both the forward and reverse strands and is reported to detect mutations as low as 10^−7^ (Schmitt et al. 2012; Kennedy et al. 2014). Note that for dPCR, we screened more molecules than for DS (approximately 6 × 10^6^ vs. approximately 0.5 to 3 × 10^5^ molecules per variant, respectively) and the DS data primarily derives from pooled measurements of 4 to 5 donors. These differences may explain the larger VAF deviations between these 2 methods, particularly for variants c.1000G > A and c.1118A > G (Table 1 and supplementary table S5, Supplementary Material online). Based on the Fisher's exact test, the average VAFs measured with dPCR or DS were significantly different for variants c.1000G > A and c.1138A > G. For all the other variants we did not observe a significant difference between average VAFs derived from these 2 different methods.

**Table 1:** Variant allele frequencies (VAF) estimated for different *FGFR3* variants in sperm DNA

			Sperm dPCR data					
		
Loci	AA	VAF-DS	Mean VAF	Median VAF	IQR	Max VAF	Max VAF/IQR	*n*
c.952G > A	p.D318N	1.8 × 10^−5^	2.2 × 10^−5^	1.5 × 10^−5^	±2.0 × 10^−5^	1.8 × 10^−4^	9	91
c.1000G > A	p.A334T	*1.0 × 10^−5^	9.1 × 10^−5^	8.5 × 10^−5^	±1.2 × 10^−4^	2.6 × 10^−4^	2	90
c.1118A > G	p.Y373C	**6.5 × 10^−5^	1.3 × 10^−5^	0	±7.8 × 10^−6^	4.8 × 10^−4^	62	98
c.1138G > A^a^	p.G380R	5.2 × 10^−5^	4.1 × 10^−5^	2.9 × 10^−5^	±3.9 × 10^−6^	2.6 × 10^−4^	66	56
c.1172C > A	p.A391E	0	8.6 × 10^−6^	0	±8.0× 10^−6^	8.1 × 10^−5^	10	79
c.1262G > A	p.R421Q	1.3 × 10^−5^	1.7 × 10^−5^	1.1 × 10^−5^	±2.0 × 10^−5^	1.6 × 10^−4^	8	99
c.1537G > A	p.D513N	0	9.4 × 10^−6^	5.0 × 10^−6^	±1.0 × 10^−5^	9.0 × 10^−5^	9	91
c.1620C > A	p.N540K	7.5 × 10^−5^	1.1 × 10^−5^	5.0 × 10^−6^	±1.8 × 10^−5^	1.0 × 10^−4^	6	108
c.1862G > T	p.R621L	0	2.7 × 10^−6^	0	±5.0 × 10^−6^	2.4 × 10^−5^	5	93
c.1879G > A	p.E627K	9.0 × 10^−5^	1.5 × 10^−5^	1.1 × 10^−5^	±1.4 × 10^−5^	8.0 × 10^−5^	6	79
c.755 C > G^b^	p.S252W	…	2.4 × 10^−5^	8.9 × 10^−6^	±2.4 × 10^−5^	7.3 × 10^−4^	31	314

VAF-DS represents data from duplex sequencing (Salazar et al. 2022) summarized in supplementary table S5, Supplementary Material online.

*P-value = 2x10^−4^ and **P-value = 2x10^−2^ (Fisher's exact test, FET) estimated between DS and dPCR. VAF-dPCR are mean VAF values estimated from data of individual sperm donors (supplementary table S4, Supplementary Material online), the same applies to maximum VAF (Max VAF). Taken from ^a^Striedner et al. (2024) and ^b^Yoon et al. (2009), the latter reporting VAFs for the c.755C > G variant in *FGFR2* associated with Apert syndrome.

AA, amino acid; VAF, variant allele frequency; IQR, interquartile range.

### Increase of VAFs with Donor's Age

**Fig. 3.**
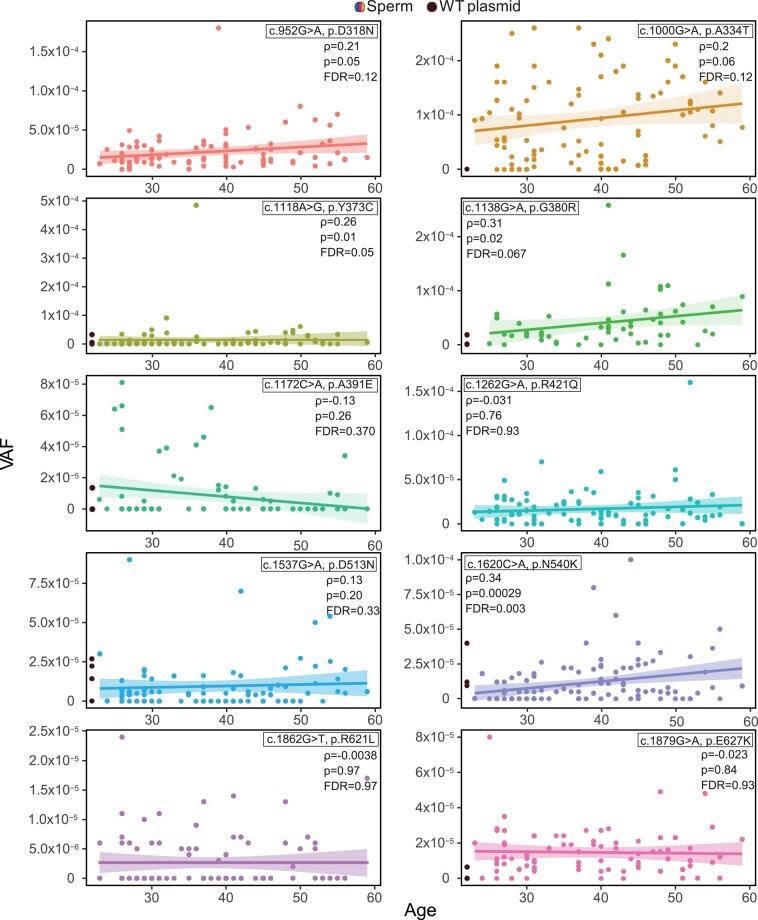
Analysis of *FGFR3* variants in sperm DNA. Mutation frequency of *FGFR3* variants measured in sperm DNA donors is listed in supplementary table S4, Supplementary Material online. Variants c.1118A > G and c.1138G > A were measured with the in-house PCR approach; data for c.1138G > A were taken from Striedner et al. (2024). For comparison, WT plasmid (black circles next to the *y* axis) were also plotted if available. The correlation of VAF with age was estimated by the Spearman’s correlation coefficient (*ρ*). We also report both the unadjusted *P*-value (*P*), and the FDR (computed by the Benjamini and Hochberg method) which adjusts for multiple tests.

We assessed if there was an increase in mutations with age by the Spearman's correlation coefficient (*ρ*), and tested the significance of this correlation. In Fig. 3, we report both the unadjusted *P*-value (*P*) and the false discovery rate (FDR, computed by the Benjamini and Hochberg method) which adjusts for multiple tests. The individual VAF measurements reported for each sperm donor can be found in supplementary table S4, Supplementary Material online.

The strongest correlation between age and VAF was observed for variant c.1620C > A with the Spearman's correlation test indicating a significant increase with paternal age (*ρ* = 0.34; FDR < 10^−2^). Further, for this variant the average VAFs were significantly different among age groups when comparing younger (≤30) with middle (31 to 44) and older (≥45) donors (supplementary fig. S2, Supplementary Material online). We also observed a similar positive correlation in VAF with age for variant c.1118A > G (*ρ* = 0.26, FDR = 0.05). Also the canonical PAE variant (c.1138G > A) was reported to increase with paternal age (Striedner et al. 2024) with a similar Spearman's correlation value as the previous variants (*ρ* = 0.31; FDR = 0.067), but bordered on significance after the multiple tests adjustment. Variants c.952G > A (*ρ* = 0.21) and c.1000G > A (*ρ* = 0.2) also showed a positive correlation of VAF with age, but this correlation was not significant (FDR = 0.12 and 0.12, respectively). Note that the VAF was independent of other factors such as sperm diagnosis or sperm count (million sperms/ml) and no correlation was observed when testing the 177 screened donors (supplementary table S6, Supplementary Material online).

In comparison, 3 other variants categorized as pathogenic or likely benign and/or uncertain significance by ClinVar (c.1172C > A, c.1537G > A, and c.1879G > A) did not show an increased VAF with age (Fig. 3). Further, the VAFs were similar across the different age categories (supplementary fig. S2, Supplementary Material online). Notably, we observed donors with high VAFs ∼1 × 10^−4^, but these were independent of age with mostly younger or middle-aged donors carrying this mutation (Table 1).

For comparison purposes, we included in Table 1 published datasets, including variant c.755C > G of *FGFR2* (associated with Apert syndrome) that was screened also with a dPCR approach in a large number of sperm donors at a depth of 1 million genomes, and robust validation (Yoon et al. 2013). Additionally, it is a valuable reference point for studying the PAE, drawing from incidence data (Risch et al. 1987), sperm, and testis dissection data (Qin et al. 2007). Our study observed individual donors reaching high VAFs (e.g. ∼5 × 10^−4^; Table 1) and the MaxVAF differed from the interquartile range (IQR) by a factor of 5- to 66-fold, except for c.1000G > A (Table 1). This supports that the lack of a positive correlation with the donor’s age for some variants is not due to assay limitations.

### Characterization of Novel Variants

Three uncharacterized variants c.1262G > A (p.R421Q), c.1862G > T (p.R621L), and c.952G > A (p.D318N) had a high predicted deleteriousness score, and were reported at higher frequencies in sperm DNA (Salazar et al. 2022). For the first variant, c.1262G > A, we observed VAFs as high as 1.6 × 10^−4^, similar to the levels of the canonical PAE variant. Yet, our data showed neither a correlation between VAF and age (Fig. 3) nor a difference in frequency between the 3 age groups (supplementary fig. S2, Supplementary Material online). The second variant, c.1862G > T, has been reported in the literature with a different amino acid substitution at the same codon (c.1862G > A, p.R621H) as a pathogenic variant with a slightly higher CADD score (29.8) associated with CATSHL (camptodactyly, tall stature, and hearing loss) syndrome (Toydemir et al. 2006). For this variant, we did not measure a positive correlation with the sperm donor's age (Fig. 3). Finally, the third variant c.952G > A has one of the lowest predicted CADD scores and a positive correlation in VAF with age (not significant).

### Spatial Distribution of Variants in the Aging Human Testis

**Fig. 4.**
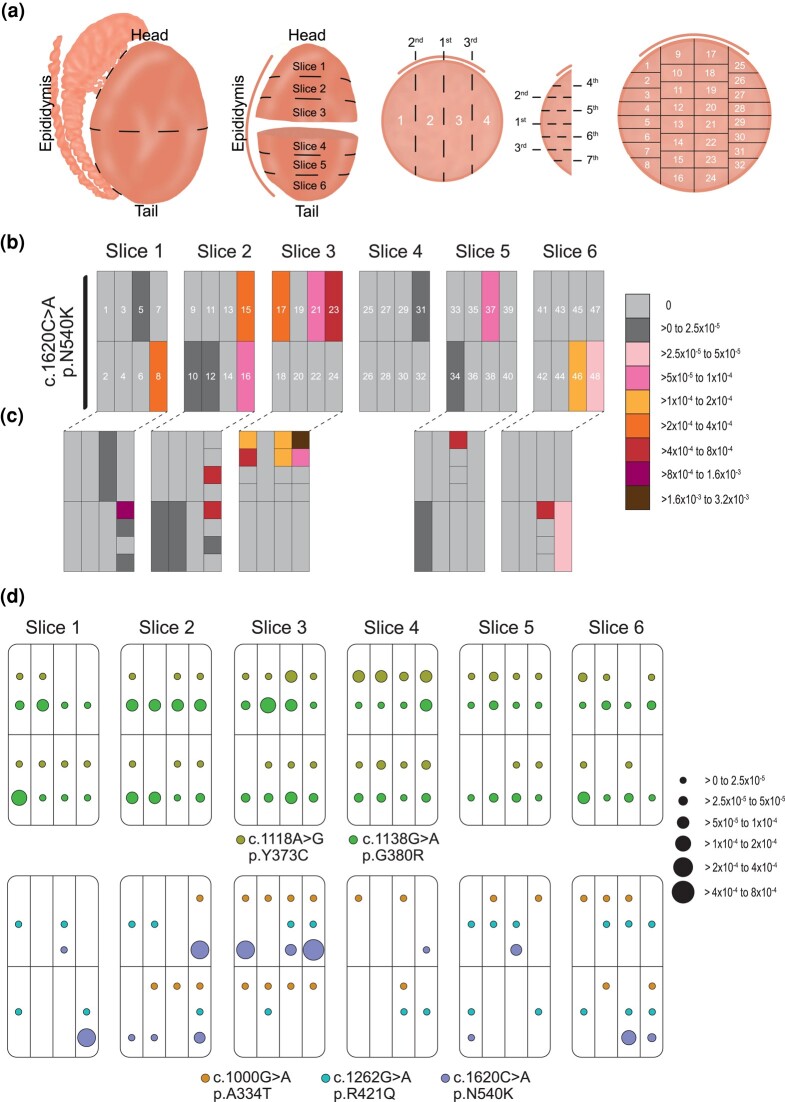
Testis data. a) Testis cutting scheme strategy. The epididymis was removed, the testis was cut in half and fixed in 70% ethanol. Each half was further cut into 3 slices as shown. Each individual slice was cut into 4 vertical strips and 7 horizontal cuts. In sum, each slice was dissected into 32 individual pieces as shown above. b and c) Mutation distribution of variant c.1620C > A in human testis of a 73-year-old donor. b) Data analysis of individual pool samples (each consisting of 4 individual adjacent testis pieces). c) In-depth analysis of “hot” pools (no. 8, 15, 16, 17, 21, 23, 37, and 46) with a mutation frequency above 5 × 10^−5^ to 1 × 10^−4^ (pink). d) Mutation distribution of variants c.1118A > G, c.1138G > A, in the testis of a 68-year-old donor, and c.1000G > A, c.1262G > A, and c.1620C > A in the human testis of a 73-year-old donor with the circle size relative to the VAF. Data of pooled samples (each consisting of 4 individual adjacent testis pieces) separated for 2 variants c.1138 and c.1118 measured using in-house PCR rather than ddPCR, and in a different testis. Empty fields represent measurements with no mutations. The data can be seen in supplementary table S7, Supplementary Material online.

Clonal expansions in solid tissues can result in high VAFs in constrained spatial neighborhoods. We therefore sought to spatially assess VAFs of 5 variants in the testis of postmortem donors (68 or 73 years old) to better understand if the observed VAFs are likely driven by clonal expansions. For this purpose, we adapted the testis microdissection technique commonly used in combination with single-molecule PCR screening (Qin et al. 2007; Choi et al. 2008, 2012; Shinde et al. 2013; Yoon et al. 2013; Striedner et al. 2024). Briefly, the human testes were dissected into 6 slices and further cut into 192 pieces (Fig. 4a).

We first assessed different pooling strategies and evaluated these in detail for the c.1620C > A variant: (i) coarse analysis: pools of 4 adjacent testis pieces per sample versus (ii) fine analysis: assessment of each individual piece (Fig. 4b and c). Mutation frequencies in the coarse analysis closely matched the mean of the fine analysis, falling within the respective SD across the 8 tested pools (supplementary fig. S3, Supplementary Material online). As a result, we measured the VAF of 48 pools merged from 192 individual pieces. For those pools with VAF > 5 × 10^−5^, we measured in addition the 4 individual pieces.

We illustrated in Fig. 4d the spatial distribution of 5 variants: the canonical mutation c.1138G > A and other 4 variants (data in supplementary table S7, Supplementary Material online, and in Striedner et al. 2024 for variant c.1138G > A). The selection of these other variants was based on (i) the variants showing a positive correlation of age with VAF (c.1118A > G, c.1620C > A and potentially c.1000G > A) and (ii) an uncharacterized variant with no increase with age and no association with any phenotypes, but a high VAF and high CADD score (c.1262G > A). We observed that for the canonical variant, some testis pieces measured VAFs >5 × 10^−5^ next to pieces with lower VAFs or no mutations (Fig. 4d). Supplementary table S7, Supplementary Material online shows that individual pieces with VAFs ∼10^−4^ or 10^−3^ within the pool mostly contributed to this signal. Similarly, c.1620C > A was highly clustered with the piece harboring the largest VAF being adjacent to pieces with a much lower or absent frequency (Fig. 4b to d, and supplementary table S7, Supplementary Material online).

We introduced several summary statistics to better assess the amount of mutation clustering (Table 2). For the canonical PAE variant c.1138G > A, we measured mutations in all 48 pools (average frequency of 3.5 × 10^−5^); yet, some pools were much hotter than neighboring ones. The highest incidence piece/pool had a VAF of 4.1 × 10^−4^, representing a ∼13-fold increase from the IQR (Max/IQR; Table 2). In the case that mutations are uniformly distributed (no cluster formation), the Max/IQR should be closer to 1. For variant c.1620C > A, the ratio of the maximum VAF in a piece to the rest of the testis (Max/IQR) differed by more than 3 orders of magnitude (1800-fold). This is an indication of a very strong clonal expansion event, much stronger than observed for the canonical PAE mutation.

**Table 2:** Screening of 5 *FGFR3* mutations in testis DNA

Loci	*n*	Median VAF	IQR	Mean VAF	SD	Max piece	Max/IQR	*P*-value (hotspot model)	Variant expansion behavior
c.1000G > A	48	0	4.5 × 10^−6^	3.4 × 10^−6^	± 5.3 × 10^−6^	2.3 × 10^−5^	5.1	1.0E + 00	Unknown
c.1118A > G	48	4.9 × 10^−6^	2.1 × 10^−5^	1.4 × 10^−5^	± 2.0 × 10^−5^	1.5 × 10^−4^	7.3	7.5E-01	Unknown
c.1138G > A	48	3.0 × 10^−5^	3.3 × 10^−5^	3.5 × 10^−5^	± 2.4 × 10^−5^	4.1 × 10^−4^	12.9	3.9E-02*	Selective advantage
c.1262G > A	48	0	6.0 × 10^−6^	4.1 × 10^−6^	± 5.7 × 10^−6^	2.2 × 10^−5^	3.7	1.0E + 00	Unknown
c.1620C > A	48	0	4.0 × 10^−6^	3.5 × 10^−5^	± 9.7 × 10^−5^	1.8 × 10^−3^	1800	1.4E-05*	Selective advantage

Note that no individual pieces were measured for c.1000G > A and c.1262G > A (no pools with VAFs > 5 × 10^−5^). IQR, interquartile mutation frequency; Max piece, maximum mutation frequency in individual pieces or coarse pools. Max/IQR, ratio of the max piece or pool to testis IQR mutation frequency.

In contrast, c.1000G > A had a 10-fold lower average frequency of 3.4 × 10^−6^ in the whole testis, with a Max/IQR ratio of ∼5 (Fig. 4d, supplementary table S7, Supplementary Material online). The c.1262G > A locus had a similarly low average VAF of 4.1 × 10^−6^, with the maximum VAF being ∼4-fold higher compared to the testis IQR frequency, closer to the expected proportion in the absence of clonal expansions.

In order to investigate what causes the observed cluster formation, we incorporated a modeling framework that has been previously applied to similar measurements from testis dissection studies (Qin et al. 2007; Choi et al. 2008; Arnheim and Calabrese 2009; Choi et al. 2012; Shinde et al. 2013; Yoon et al. 2013; Arnheim and Calabrese 2016; Eboreime et al. 2022). In the first model we considered, called the symmetric hot spot model (Choi et al. 2012; Shinde et al. 2013; Yoon et al. 2013; Arnheim and Calabrese 2016; Eboreime et al. 2022), the mutated stem cells behave the same as the WT stem cells. Any cluster formation is caused simply by the normal process of stem cell division and differentiation. This model has 1 free parameter: the mutation rate per cell division.

In order to test this model, we simulated the model such that the simulated average testis mutation frequency nearly matches the observed average testis mutation frequency. For these simulations, we then compared the simulated maximum testis piece mutation frequency to the observed maximum testis piece mutation frequency (the *P*-value is the fraction of simulations where the simulated maximum testis piece mutation frequency is greater than the observed maximum testis piece mutation frequency). We described the model, and our parameter fitting and statistical testing strategies, in more detail in the Methods section. In Table 2, we can see that for 3 of the mutations (c.1000G > A, c.1118A > G, and c.1262G > A) we cannot reject the hot spot model. For these 3 mutations, the observed maximum testis piece mutation frequencies are within the range of the simulations. However, for 2 of the mutations (c.1138G > A and c.1620C > A), we can reject the hot spot model. For these 2 mutations, the observed maximum testis piece mutation frequencies are greater than almost all of the simulations.

For these 2 mutations we then considered a second model, called the symmetric selection model (Yoon et al. 2013; Arnheim and Calabrese 2016; Eboreime et al. 2022). This model is similar to the hot spot model except that the mutated stem cells behave differently than the WT stem cells. The difference is that the mutated stem cells (but not the WT stem cells) are more likely to divide symmetrically than to differentiate. This difference makes mutation clusters more likely to form. This model also has 1 free parameter: the bias between symmetric divisions and differentiation in the mutated stem cells. This model is also explained in more detail in the Methods section.

We tested this model the same way as the hot spot model (for the simulations that match the average testis mutation frequency, we compared the simulated maximum testis piece mutation frequency to the observed maximum testis piece mutation frequency). We found that for both of these mutations, we could not reject the selection model (the observed maximum testis piece frequencies were within the range of the simulations). The inferred bias for symmetric divisions versus differentiation is slight (0.5024 to 0.4976 for mutation c.1138G > A, and 0.5029 to 0.4971 for mutation c.1620 C > A). However, since this bias is shared by subsequent generations of the mutated stem cells, it is sufficient to reproduce the extreme observed mutation clusters. Also, previously (Qin et al. 2007; Choi et al. 2012; Arnheim and Calabrese 2016) we considered several other models and the selection model is the only one that we found that was consistent with the extreme observed mutation clusters.

### *FGFR3* Variants Modify the Receptor's Adaptor Protein Recruitment

**Fig. 5.**
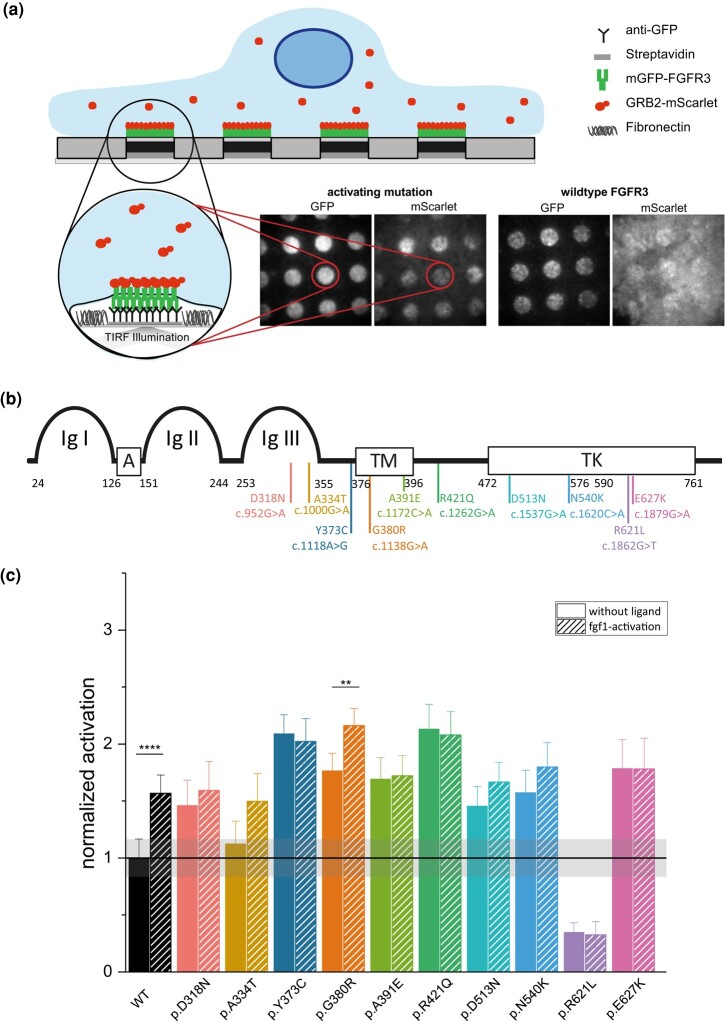
Signaling modulation by de novo *FGFR3* mutations in HeLa cells. a) Illustrative representation of measurement principle in the ligand-free state for WT FGFR3 and an activating FGFR3 mutation. mGFP-labeled FGFR3 and mScarlet-labeled GRB2 are coexpressed in HeLa cells. Plasma-resident FGFR3 is captured in specific patterns via an anti-GFP antibody leading to FGFR3-enriched and FGFR3-depleted regions. Expression of an activating mutation or FGF1 addition to WT FGFR3 leads to the recruitment of GRB2 to the sites of FGFR3. The degree of recruitment is expressed as contrast mScarlet (C_mScarlet_). b) Schematic indicating the approximate location within the protein domains of the different variants. c) The mean C_mScarlet_ value of all candidate variants both in the nonactivated and FGF1-stimulated state was normalized to the value of the nonactivated WT. The data are shown as normalized mean values with the respective standard error of the mean of a ratio as described in Supplementary Material onlineand supplementary table S8, Supplementary Material online. The WT, p.Y373C, and p.G380R datasets (nonactivated and FGF1-activated) were previously published in Hartl et al. (2023). The mean of the nonactivated WT (black line) is shown with its confidence interval (gray area). Please note that every mean value of nonactivated datasets within the confidence area is not significantly different from the WT. The number of cells measured for each variant is listed in supplementary table S8, Supplementary Material online. Significant differences in variants before and after activation tested via pairwise ANOVA on the raw data are indicated and listed in supplementary table S9, Supplementary Material online. The *P*-value annotations are represented as follows: *P* ≤ 0.01 (**), and *P* ≤ 0.0001 (****).

Here, we sought to further investigate the activation of the studied *FGFR3* variants at the cellular level. For this, we used a protein micropatterning approach (illustrated in Fig. 5a) that allows the study of protein–protein interactions at the plasma membrane in living cells using TIRF microscopy (Schwarzenbacher et al. 2008; Lanzerstorfer et al. 2014; Schütz et al. 2017; Motsch et al. 2019). In a recent study, we have demonstrated that this approach is quite robust for studying the activation level of FGFR3 when measuring the interaction with the downstream adaptor protein GRB2 (Hartl et al. 2023). Here, we coexpressed each of the different FGFR3 variants (labeled with mGFP) with GRB2 (labeled with mScarlet). In the plasma membrane, mGFP-FGFR3 is enriched and immobilized within certain areas that are defined by an antibody pattern on the cover slip. If FGFR3 is activated, the kinase domain gets phosphorylated and interacts with downstream signal adaptors such as GRB2-mScarlet-I and both fluorophore-tagged proteins colocalize within the same micropatterned areas (Fig. 5a). The degree of activation is proportional to the level of GRB2 recruited to active FGFR3, given here as the normalized mScarlet contrast, which relates the signal inside and outside the mGFP-FGFR3-enriched areas (see Methods section for details). Figure 5b shows the approximate location of the analyzed variants on the protein. The 2 canonical PAE-mutations c.1118A > G (p.Y373C) and c.1138G > A (p.G380R) were previously characterized with the same technique (Hartl et al. 2023).

Both FGFR3 mutations p.Y373C and p.G380R lead to increased receptor signaling already in the absence of ligand compared to the WT. Moreover, p.G380R could be further activated by FGF1 addition (supplementary table S8, Supplementary Material online). Of the other 8 variants, 2 showed similar or lower activity than the WT receptor. The p.A334T (c.1000G > A) mutation located in the Ig-III extracellular domain of FGFR3 did not show a different receptor activity than the wild type (Fig. 5c and supplementary table S8, Supplementary Material online), whereas p.R621L (c.1862G > T) located in the kinase domain in the C-lobe of the split tyrosine kinase domain (Farrell and Breeze 2018; TK2 in Fig. 5b) significantly inhibited the receptor activity.

The remaining 6 variants lead to a significant increase in receptor signaling in the nonliganded state compared to the WT receptor (Fig. 5c). These variants (c.952G > A, p.D318N, Ig-III domain; c.1172C > A, p.A391E, TM domain; c.1262G > A, p.R421Q; c.1537G > A, p.D513N, TK1 domain; c.1620C > A, p.N540K, TK1 domain and c.1879G > A, p.E627K, TK2 domain) presented a high activity with a 1.5- to about 2.5-fold increase in mScarlet contrast already in the absence of ligand which is comparable to the level of the ligand-activated WT receptor (Fig. 5c). The difference in activation between WT and these 6 mutants was significant (supplementary table S9, Supplementary Material online).

Notably, only 3 out of these 6 variants have a reported deleterious score in ClinVar (p.A391E, p.D513N, and p.N540K). However, variant p.R421Q located in the intracellular juxta-membrane region of the receptor has not been described in ClinVar and has the highest CADD score; yet, according to SIFT metrics it is at the borderline for being tolerated (0.06). It also had the strongest promiscuous receptor activation (without ligand addition) compared to the other analyzed variants. None of these 8 novel variants were further activated by the addition of the cognate FGFR3 ligand FGF1. These results show that in silico annotations like the CADD or SIFT score are not always accurate in predicting the biochemical effect of the mutation. Variants with similar CADD scores had different levels of ligand-independent activation, as seen in the extreme case for variant c.1862G > T (p.R621L) that resulted in a loss-of-function compared to other high CADD score variants. Also, variants predicted to be tolerated based on sequence conservation (SIFT score) such as p.Y373C, p.G380R, p.R421Q, and p.D513N did significantly alter the receptor activation in the absence of ligand.

## Discussion

With our unique sequence-functional analysis, we provide an overview of the landscape of expansions of different *FGFR3* variants in the male gonad. We found that 9 out of 10 of the analyzed *FGFR3* variants are gain-of-function with promiscuous activation (ligand independent). Some variants increased in frequency with age in sperm and also formed larger clusters in the male gonad. However, other variants were present already at high levels in sperm of young individuals and did not expand to measurable clusters with age. This suggests 2 types of mutational trajectories: one that grows to larger subclonal clusters in the sexually mature gonad and increases in frequency in sperm with age (e.g. canonical PAE mutations). The other type likely occurs prepuberty forming stable niches that stay constant in size. As a consequence, the incidence of mutations forming gonadal micromosaics with potential health consequences in the offspring might be much more common in the population and in some cases independent of the age of conception.

### Use of dPCR in the Detection of Rare Variants

We used dPCR, a method less sensitive than DS, but with the careful preparation and sample handling, it is capable of measuring VAFs at ultralow levels in hundreds of samples. The discrete compartments (either beads or droplets) isolate single DNA molecules making the dPCR method more sensitive than other qPCR-based methods with larger mixtures of molecules susceptible to unspecific binding of primers or probes as used previously (Tiemann-Boege et al. 2002; Goriely et al. 2003). The in-house dPCR approach (BEA) (Tiemann-Boege et al. 2009; Shinde et al. 2013) demonstrated similar levels of sensitivity and accuracy compared to ddPCR as reported in Striedner et al. (2024). However, both approaches are susceptible to background noise levels coming from false mutation calls. PCR errors occurring in the first amplification cycle could contribute to these false mutant counts. Further, unlike methods that analyze both DNA strands such as DS (Schmitt et al. 2012; Kennedy et al. 2014), dPCR only analyzes one of the DNA strands. Thus, dPCR is also limited in sensitivity by DNA lesions. Cytosine deamination or guanine oxidation are the most common lesions that can occur during DNA extraction using prolonged heating steps or multiple freezing/thawing cycles that result in false mutation calls at levels of ∼10^−6^ or higher (Arbeithuber et al. 2016).

Given the large number of molecules we sampled per site, we hypothesized that the contribution of PCR errors or lesions to the false signals should be reflected in sperm samples without a mutation count. Samples without mutations summed to 10^6^ to 10^7^ molecules. This is an order of magnitude higher than the WT genomes we used as input per sample and we conclude that the signal from lesions (or false positives) is negligible in our positive samples with VAFs > 10^−5^.

### Phenotype of Novel Variants

Several studies have focused on the signaling effects of receptor tyrosine kinase (RTK) mutations (Naski et al. 1996; Wilkes et al. 1996; Li and Hristova 2006; Toydemir et al. 2006; He and Hristova 2008; Foldynova-Trantirkova et al. 2012; Ornitz and Itoh 2015). Growth factor availability is tightly controlled in the body via various mechanisms, as well as the interaction between FGFs and the extracellular matrix that influences the receptor affinity and diffusion of ligands through the tissue (Flaumenhaft et al. 1990; Ornitz 2000). If an amino acid change is highly activating and independent of ligand binding, this might have the outcome of cells constantly receiving growth signals and lead to a growth advantage and clonal expansion typical of driver mutations. Even though there are many in silico tools that predict the deleteriousness of a protein's structure, the modification on the downstream signaling is unknown and can have different signaling outcomes. This was the case of the uncharacterized variants c.1262G > A (p.R421Q), c.1862G > T (p.R621L), and c.952G > A (p.D318N), all with high CADD scores, different pathogenic SIFT scores, and different signaling outcomes.

Variant c.1262G > A (p.R421Q) is located in the intracellular juxta-membrane region of FGFR3 and has the highest predicted deleteriousness score, as well as the highest signaling activation detected with micropatterning, irrespective of FGF1 addition. Despite the fact, that c.1262G > A (p.R421Q) is not located in the catalytically active domain of the receptor, it resulted in the highest activation. Notably, this variant behaves similarly to the c.1879G > A (p.E627K) mutation in terms of activation and cellular effect (similar mutation frequency and number of positive donors in sperm). Yet, the p.E627K variant is quite close to the catalytically active residue, in the C-lobe of the kinase domain, explaining the second-highest activation. These findings indicate that the location of the mutation in the protein sequence is independent of the impact on the signaling output.

The second novel variant, c.1862G > T (p.R621L) showed a significantly decreased GRB2 recruitment compared to the WT receptor both in the nonliganded and the FGF1-activated state, similar to the established negative control (Hartl et al. 2023). This variant has never been described in the literature and has no associated syndromes. Although this mutation has one of the highest CADD scores, it has the lowest mutation frequency, and it is only found in 34% of donors. This variant actually decreases the receptor activity and therefore is possibly transmitted less in the germline. In line with our findings, a mutation in the same amino acid (p.R621H) has been reported to have a loss-of-function phenotype (Toydemir et al. 2006). The close proximity of the R621 amino acid to the invariant residue D618, which catalyzes phosphorylation within the conserved HRD motif (Farrell and Breeze 2018), possibly explains the loss-of-function phenotype associated with mutations in this position.

The third novel variant c.952G > A (p.D318N) is located in the extracellular Ig-III domain of the receptor. The mutation leads to a low significant increase in GRB2 recruitment in the nonactivated state as measured with micropatterning. The activation mechanisms might be explained by an altered affinity to FGF1 given that the ligand binding in FGFR family members is regulated by the Ig-II and Ig-III domains as well as the linker region in between these domains (Ornitz and Itoh 2015). Further, we observed a positive increase in VAF with age. This variant has not been associated with any syndromes and has a low predicted deleteriousness. Thus, we speculate that the overactivation from this variant is well tolerated and might also clonally expand with age. Spatial distribution analysis in the testis of the p.D318N variant would further our knowledge of this hypothesis. Some parallels for c.952G > A (p.D318N) can be made with the mildly pathogenic c.1537G > A (p.D513N) mutation, associated with the Levy–Hollister syndrome. The Levy–Hollister syndrome has been reported as a unique case as it phenotypically differs from both gain-of-function associated congenital disorders (chondrodysplasias) and loss-of-function syndromes (skeletal over-growth and hearing loss) (Toydemir et al. 2006; Horton et al. 2007; Ornitz and Marie 2015). We measured for both variants one of the lowest activation levels in the nonliganded state and after the addition of FGF1. However, unlike p.D318N, the ddPCR results showed no significant enrichment in sperm with increasing age for variant p.D513N and was found in a lower number of donors (60% instead of 93%).

### Activating *FGFR3* Substitutions and Subclonal Expansions in the Male Germline

Larger subclonal clusters have been previously reported in the aging testis for mutations associated with Apert syndrome (Qin et al. 2007; Choi et al. 2008, 2012; Maher et al. 2016), Noonan syndrome (Yoon et al. 2013; Maher et al. 2018; Eboreime et al. 2022), Multiple Endocrine Neoplasia Type 2B (MEN2B) (Choi et al. 2012), Thanatophoric Dysplasias (Maher et al. 2016), ACH (Shinde et al. 2013), Pfeiffer syndrome (Maher et al. 2016, 2018), Hypochondroplasia, Crouzon, MEN2A, and Beare–Stevenson syndromes (Maher et al. 2018).

These mutations are hypothesized to expand with age in the sexually mature male gonad by a selective advantage conferred by the mutant protein and are classified as PAE mutations. The PAE and high mutation rate are likely associated with a dysregulation of spermatogonial stem cells, in this case actively dividing (SrAp) (Goriely et al. 2003; Qin et al. 2007; Choi et al. 2008, 2012). The mutation arises rarely but expands clonally in the sexually mature testis leading to a relative enrichment of mutant spermatogonia or sperm equivalents explaining the high mutation frequencies with older age. Actively dividing SrAp normally divide asymmetrically resulting in a daughter cell and a cell that differentiates into sperm. In contrast, mutant SrAp might occasionally divide symmetrically resulting in 2 daughter stem cells leading to a progressive clonal expansion of mutant germ cells (reviewed in Arnheim and Calabrese 2009, 2016). If a mutation occurs in a multiplying colony with cells staying in close proximity, the mutation forms a cluster or subclonal expansion, with the size of the cluster depending on the number of cell divisions since the mutation occurred (Crow 2012).

Although there is no proof of causality between activation and expansion in the male germline, important patterns can be observed also in our data that are summarized in Table 3 providing new insights into the behavior of these mutations.

**Table 3:** Summary of variant behavior in terms of the maximum VAF found in sperm samples, sperm VAF increasing with the donor's age, percentage of donors with mutations in sperm, clustering in the testis, activation of FGFR3 relative to the wild type plus its response to ligand activation, and the clonal expansion model as defined in fig. 5

Significance (ClinVar)	Target site	CpG site	AA	SIFT	Max VAF	Sperm increase	Fraction of mutant sperm	Testis clustering	Fold FGFR3 activation	Activation state	Clonal expansion model
.	c.952G > A	GCG	p.D318N	0.01	1.8E-04	Positive?	93%	NA	1.5	Intermediate Ligand independent	PAE
Uncertain	c.1000G > A	CGC	p.A334T	0.03	2.6E-04	Positive?	88%	Weak clustering	1.1	Low/Physiologic	PAE?
Likely pathogenic	c.1118A > G	…	p.Y373C	0.09	4.8E-04	Positive	46%	Subclonal expansion?	1.8	High ligand independent	PAE?
Pathogenic	c.1138G > A	CGG	p.G380R	0.09	2.6E-04	Positive?	91%	Subclonal expansion	1.7	High ligand dependent	PAE
Pathogenic	c.1172C > A	…	p.A391E	0.04	8.1E-05	No correlation	32%	NA	1.7	High ligand independent	Age independent
.	c.1262G > A	CGA	p.R421Q	0.06	1.6E-04	No correlation	82%	Weak clustering	2.1	High ligand independent	Age independent
Pathogenic/Uncertain	c.1537G > A	CGA	p.D513N	0.63	9.0E-05	No correlation	60%	NA	1.5	Intermediate ligand independent	Age independent
Pathogenic	c.1620C > A	…	p.N540K	0	1.0E-04	Positive	63%	Subclonal expansion	1.6	Intermediate ligand independent	PAE
.	c.1862G > T	…	p.R621L	0	2.4E-05	No correlation	34%	NA	0.4	Inhibited	Unknown
Likely benign/Uncertain	c.1879G > A	CGA	p.E627K	0.03	8.0E-05	No correlation	89%	NA	1.8	High ligand independent	Age independent

AA, amino acid; SIFT, sorting intolerant from tolerant score (≤0.05 is considered deleterious); VAF, variant allele frequency; TK, tyrosine kinase; TM, transmembrane; Ig-III, immunoglobulin-like domain III; NA, not analyzed; PAE, paternal-age effect.

First, variants increasing in frequency with the sperm donor's age like c.952G > A, c.1118G > A, c.1138G > A, and c.1620C > A (albeit some being nonsignificant after FDR correction) also showed a ligand-independent FGFR3 activation, and likely form larger expansion clusters in the aging testis. In fact, c.1620C > A (p.N540K) showed the largest increase in VAF with the donor’s age and the strongest support for subclonal cluster formation in the dissected testis. A high VAF in sperm for this variant, together with a second substitution (c.1620C > G), resulting in the same amino acid change (p.N540K) was reported using DS (Salazar et al. 2022). Enrichment of c.1620C > G and c.1620C > A was also reported in testis biopsies sampled for FGFR3 activation (Maher et al. 2018). This suggests that both variants c.1620C > A and c.1620C > G (same amino acid change resulting in promiscuous signaling) increase with age in the male germline. Notably, the c.1620C > A (p.N540K) mutation has been described as the most prominent mutation causing hypochondroplasia (Bellus et al. 1995; Rousseau et al. 1996; Prinster et al. 1998; Ramaswami et al. 1998; Xue et al. 2014), although other congenital disorders such as ACH, TDI (Pannier et al. 2009), and Craniosynostosis associated with variant c.1620C > A (p.N540K) have also been reported in ClinVar.

Variant c.1118A > G (p.Y373C) is one of the most predominant causal mutations of TDI (Rousseau et al. 1996). It has been reported to increase the incidence of affected children with paternal age, as shown in studies of hospital births (Orioli et al. 1986). This work presents further evidence for an increase with the sperm donor's age. An enrichment of this variant was also observed with DS in sperm DNA of older donors (Salazar et al. 2022). Given the high ligand-independent activation caused by this substitution, it is likely that subclonal expansion is promoted mainly by the positive selection of actively dividing mutant SrAp in the sexually mature testis. However, the evidence for clonal expansions in the aging male gonad for this variant was not statistically significant, despite the high VAFs observed in individual testis pieces. Potentially a finer pooling strategy might provide more conclusive results.

The canonical PAE variant c.1138G > A showed both an increase in frequency with age, and subclonal clusters in the aged testis, as reported previously (Tiemann-Boege et al. 2002; Shinde et al. 2013; Salazar et al. 2022; Striedner et al. 2024). However, in comparison to the findings in Shinde et al. (2013), the evidence for subclonal expansion in the testis was less robust (see Table 2), possibly due to the use of a broader pooling strategy. Variant c.952G > A also showed an increase in VAF with age (albeit nonsignificant after FDR correction). Whether this variant also forms measurable subclonal clusters in the aged testis remains to be tested. We hypothesize that all 4 variants (c.952G > A, c.1118G > A, c.1138G > A, and c.1620C > A) expand in the sexually mature germline forming larger clusters with age associated with higher mutation frequencies measured in sperm of older donors and fall within the definition of a classical PAE.

Second, FGFR3 activation did not necessarily lead to a positive correlation of VAFs with age or testicular clustering. This was the case for variants c.1172C > A, c.1262G > A, c.1537G > A, c.1879G > A (Table 2). Variant c.1172C > A had the lowest pathogenicity scores of our candidate mutations, but is associated with Craniosynostosis and Crouzon syndrome with Acanthosis Nigricans, both described to increase in incidence in offspring of older fathers (Risch et al. 1987; Stoll et al. 1989). Despite the increased activating capabilities of the receptor, this variant showed the lowest number of positive donors (32%) of all analyzed mutations. One could speculate that the activation of this variant still increases cell proliferation, but might be tolerated only at very low levels. Spatial distribution analysis in the testis would be insightful in this regard.

Variant c.1262G > A had one of the highest constitutive activation states (∼2.5 × activation) but showed no correlation in VAF with the donor's age, and no measurable evidence for clustering in the aged testis. Finally, the expansion patterns of c.1000G > A show yet another trend. Sperm VAFs were high for most donors (with a slight increase with age bordering on significance), but no measurable clusters formed in the aged testis. In addition, this substitution had hardly an effect on the receptor signaling compared to the wild type.

**Fig. 6.**
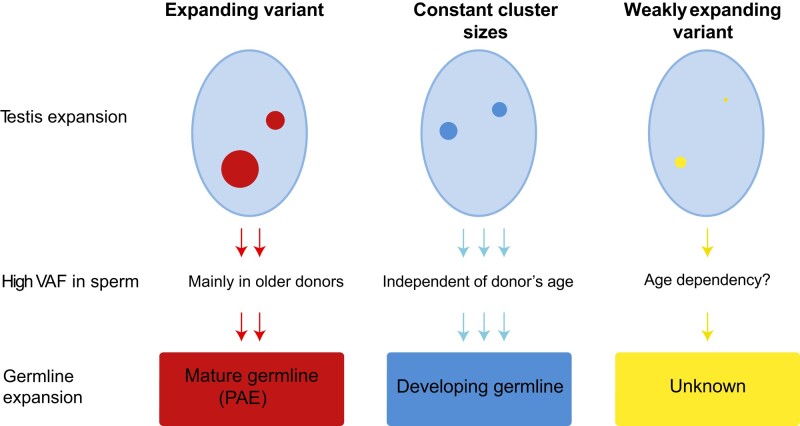
Clonal expansion in testes of aging men and the proposed expansion dependency with age by the mutant receptor.

Based on these observations, we propose that activating mutations in *FGFR3* might have an overall selective growth advantage, but do not follow the same clustering patterns in the aged testis. Some variants expand in the mature germline, whereas others might reach high levels already before sexual maturation of the male germline with the VAF staying relatively constant in the aging gonad (Fig. 6).

### Age-Dependency of Clonal Expansion Events

During development, a series of random *DNMs* arise at any given time point in different cell lineages that change in number driven by different evolutionary processes. Some mutations might be linked to selection (Martincorena et al. 2018), others by the stochastic process of genetic drift (McFarland et al. 2014). The deleterious activating effect of the mutation might also have a negative outcome hindering cell fitness and decreasing the risk of transmission or might be tolerated only at very low levels (reviewed in Tiemann-Boege et al. 2021).

The chance of encountering any particular *DNM* at a given specific site is very small and only measurable with high-accuracy methods that capture 1 specific nucleotide substitution within large populations of cells (>10^6^) in many individuals. Our study showed that in most variants, 60% to 90% of the sperm donors harbored a measurable number of mutations (with individual samples reaching VAFs as high as ∼5 × 10^−4^). This large proportion of individuals with *DNMs* could be explained by: (i) our variants being hypermutable sites and (ii) mutations occurred already throughout development and increased to measurable levels in the male gonad by a selective advantage.

A “hypermutable site” explanation is not very satisfying for missense coding variations. But how can a selective advantage explain a high *DNM* rate and an age-independent expansion? We suggest that mutations might also arise and expand before the male germline becomes sexually mature. The accumulation of mutations in the germline during developmental phases has been observed also in other species (de Manuel et al. 2022). A similar observation was made for genome-wide mutations, with a small fraction of *DNM* being shared among siblings, suggesting that these must have accumulated throughout development forming mosaics in the father's germline (Rahbari et al. 2016; Sasani et al. 2019).

The question is, what is the fate of activating mutations originating before the sexually mature gonad linked to a selective advantage? Will these expand to very large clusters in the aging gonad? The data for c.1000G > A and c.1262G > A suggest otherwise, showing minimal expansions in the aging testis. A recent report on sperm mosaics also identified early developmental clones that were temporally stable across serial samples and age groups with no changes in size (or frequency) in the stem cell niches (Yang et al. 2021). A possible explanation of this constancy in size might be that some variants are only tolerated at low levels as is often observed for highly activating rasopathies forming mosaics in the skin (reviewed in Tiemann-Boege et al. 2021).

The idea that mutations can already accumulate in the germline throughout development would support the age-independency of the high VAFs we measured for some variants. Given the extensive role of FGFR receptors in many developmental processes, it is likely that the expansion of driver mutations is more complex than assumed and not just the result of selective attributes gained by the stem cell in the sexually mature germline, but rather the interplay of several biological pleiotropic mechanisms affecting spermatogenesis all the way to fertilization, with some effects being antagonistic.

## Methods

### Sample Collection

Sperm samples were collected from anonymous donors by the Kinderwunsch Klinik, MedCampus IV, Kepler Universitätsklinikum, Linz, according to the ethical approval (F1–11). Donors were aged from 23 to 59 years of age and mostly of European ancestry. Buccal samples were collected from an anonymous female donor aged 25 years old by swab collection. Cultured B-lymphocyte cells encoding the c.1620C > A variant in *FGFR3* (GM18666) were purchased from Coriell Institute (Camden, NJ). Human genomic DNA (NA08859) encoding the *FGFR3* c.1138G > A mutation.

### DNA Extraction from Sperm Samples

Genomic DNA from sperm samples was extracted using the Puregene Core Kit A (QIAGEN, #1042601) according to the manufacturer's instructions with minor modifications previously described in Arbeithuber et al. (2015). In brief, DNA was extracted from a saliva swab or 25 µl of sperm (∼10^6^ sperm cells) with the addition of 0.5 µl of Proteinase K solution (20 mg/ml) and 6 µl of 1 M DTT, followed by an overnight incubation step at 37 °C. During DNA precipitation, 0.25 µl of glycogen solution (QIAGEN, #1045724) were added. Vortexing steps were replaced by intensive manual shaking for 1 min.

### DNA Extraction form Cultured Cells

DNA extraction from cultured cells (GM18666) was carried out according to the manufacturer's instructions using the Puregene Core Kit A (QIAGEN, #1042601). Initially, 200 µl of cultured media (∼2 × 10^6^ harvested cells) were spun down for 5 s at 13,000 × *g*. The supernatant was discarded, and the cell pellet was vortexed and resuspended in the remaining 20 µl of residual fluid. The cells were then resuspended at a high vortexing speed for 10 s in 300 µl of Cell Lysis solution to promote cell lysis. Following this, 100 µl of protein precipitation solution was added and the sample was vortexed for an additional 20 s at high speed. The supernatant was transferred into a fresh tube containing 300 µl of isopropanol and was gently inverted 50 times, followed by a centrifugation step at 13,000 × *g* for 1 min. The supernatant was discarded, and the isolated DNA pellet was gently washed in 300 µl of 70% ethanol and then centrifuged at 13,000 × *g* for 1 min. The dried DNA pellet was then resuspended in 100 µl of DNA hydration solution.

### Dissection of Testis

Testis (ID: NRD#ND10354) was acquired from the National Disease Research Interchange (NDRI, Philadelphia, PA) and procured by the same entity 5.7 h after the time of death. Snap-frozen, postmortem testis from a 73-year-old anonymous Caucasian donor with no precedents of alcohol, tobacco, or drug use was used in this study. The donor had no history of diabetes, chemotherapy, radiation, infectious diseases, or drug prescriptions. For the analysis of the 2 variants c.1138G > A and c.1118A > G, we used a snap-frozen, postmortem testis (NDRI, Philadelphia, PA) from a 68-year-old donor free of a history of tobacco, alcohol, and drug use. The time of death after procurement was a maximum of 6 h. The donor did not have a history of cancer, chemotherapy, and radiation. Human genomic DNA (NA08859) encoding the *FGFR3* c.1138G > A mutation was purchased from Coriell Cell Repositories (Camden, NJ).

In brief, each testis was initially thawed overnight on ice at 4 °C. The epididymis was removed, and the testis was cut in half and fixated for 72 h in 70% ethanol at 4 °C. The testis was dissected into a total of 6 slices, each of them further cut into 32 pieces as previously described (Qin et al. 2007; Choi et al. 2008; Shinde et al. 2013). For further information about the cutting strategy, see Fig. 3a.

### DNA Extraction from Testis

DNA extraction from testis was carried out for each individual testis piece using the NucleoMag Tissue Kit (Macherey-Nagel, #744300.1) according to the manufacturer's conditions except for a few modifications. In a round bottom tube, up to 20 mg of tissue immersed in 100 µl of Buffer T1 and with the aid of a 5 mm steel bead (QIAGEN, #69989) was homogenized in the TissueLyser (QIAGEN) at 25 Hz for 1 min and then briefly spun down. The steel bead was carefully removed and 100 µl of Buffer T1 and 25 µl Proteinase K solution (28.85 mg/ml) were added and mixed well prior to overnight tissue lysis at 37 °C.

### Preparation of Control Samples

We also examined WT plasmid (see WT DNA plasmid section in Supplementary material online for details). For site c.1537G > A, ∼250 ng of *Escherchia coli* carrier DNA was added to the 100,000 genome copies of WT plasmid to have a similar number of molecules and bulk DNA compared with the sperm DNA. Since no differences were observed (data not shown), *E. coli* DNA was not added in further experiments.

Positive controls were prepared by using either commercially available cell lines containing the mutation of interest or by producing plasmids containing our mutation of interest by site-direct mutagenesis—see Site-Directed Mutagenesis (SDM) section in Supplementary material online. These modified plasmids or cell lines were used in spike-in serial dilutions experiments (1:10 to 1:10,000; Fig. 1b) with a total input of ∼20,000 genomes, containing both commercially available human genomic DNA (100 ng/µl; ClonTech, #636401) and mutant DNA from either a cell line (c.1620C > A) or mutant plasmids for the remaining target sites herein characterized (primers used to produce the mutated plasmids are listed in supplementary table S10, Supplementary Material online). Three biological replicates were run for each dilution step. Additionally, for the dilution step of 1:1000, 2 PCR reactions were set up (total analyzed genomes: ∼40,000) and for the 1:10,000, 4 reactions were done (total of ∼80,000 genomes). Mutation sites c.952G > A and c.1000G > A were prepared on the pcDNA3.1 backbone containing the complete *FGFR3* ORF (Isoform IIIc). Mutations for sites c.1172C > A, c.1262G > A, c.1537G > A, c.1862G > T, and c.1879G > A were introduced in the pCR2.1 vector containing a genomic fragment of 1887bp of *FGRF3* (chr4:1804338-chr4:1806225)—see WT DNA Plasmid section in Supplementary Material online and supplementary table S11, Supplementary Material online.

### ddPCR-BioRad

The ddPCR assay design online tool (available at: https://www.bio-rad.com/digital-assays) was used to design target site-specific assays (see supplementary table S12, Supplementary Material online). For the experimental setup, standard ddPCR reaction conditions according to the manufacturer's instructions (BioRad) were used. Individual ddPCR reactions were composed of 10 µl of 10 × SuperMix for Probes (no dUTP), 6.7 µl of nucleic acid-free water, 2 µl of genomic DNA (∼125 ng/µl; ∼36,000 genomes/µl), 1 µl of probes (900 nM of each probe and 100 nM of each primer), and 0.3 µl of restriction enzyme (MseI or CviQI; 10 U/µl). Note that for 3 target sites (see supplementary table S12, Supplementary Material online), 0.05 µl of USER enzyme were added and incubated at 37 °C for 15 min. For each sample, 4 reaction replicates were set up (total of ∼300,000 genomes).

Previous to the ddPCR droplet formation, we enhanced target template accessibility by fragmenting the genomic DNA (∼300 to <10 kb) with the abovementioned restriction enzymes. In ddPCR restriction digest was carried out by incubating the reaction mix at room temperature for 15 min and then transferred into the cartridge, along with 70 µl of droplet generation oil for probes. The gasket was securely placed and the droplets were formed in the droplet generator. Approximately 43 µl of the newly formed droplet solution were transferred into a ddPCR 96-well plate and then sealed at 180 °C for 5 s in the PX1 PCR Plate Sealer (BioRad). PCR was carried out with the following conditions: 95 °C for 10 min, 40 cycles of 94 °C for 30 s, 53 to 55 °C (see supplementary table S12, Supplementary Material online for target-specific information) for 1 min, and finally a one-time step of 98 °C for 10 min. Note that a ramp rate of 2 °C/s was applied to each step and the lid was heated to 105 °C. The PCR plate was transferred into the droplet reader for an end-point data analysis using QuantaSoft Analysis Pro Software (version 1.7.4; Bio-Rad Laboratories Inc.). We determined the detection thresholds using the positive (supplementary fig. S4, Supplementary Material online) and negative controls as a guideline. We used the Poisson corrected data points in this study.

Details on the in-house dPCR method (BEA) can be found in detail in Striedner et al. (2024) and Supplementary material online, and were also published in Tiemann-Boege et al. (2009), Shinde et al. (2013), and Palzenberger et al. (2017).

### Micropatterning Experiments

Micropatterning surface preparation, activation of patterned cells, TIRF microscopy image acquisition, and microscopy data analysis were performed as previously described in Hartl et al. (2023) and Supplementary material online. Only for measurements of p.D318N, p.A334T, p.R621L, p.E627K, and another set of WT, no epoxy-coated coverslips were used. Glass slides (30 mm diameter, 1.5 mm height; Epredia, Portsmouth, NH, USA) were treated in a plasma cleaner (PDC-002 Plasma Cleaner Harrick Plasma, Ithaca, NY, USA) for 10 min followed by coating with AnteoBind Biosensor (AnteoTech, Brisbane, Queensland, Australia) for 60 min at RT to allow the streptavidin binding. Following this, a stamping procedure was performed as previously described.

### Statistical Analysis

The VAFs were estimated based on the Poisson distribution, as λ = −ln(1− (mut/(mut + wt))). The Mann–Whitney *U* and Kruskal–Wallis tests were used to test if there were differences in VAF between the different sperm diagnosis groups. Spearman’s test was used to assess the correlation between VAF and the sperm count (million sperms/ml) for each individual mutation (see supplementary table S6, Supplementary Material online). Spearman's correlation test was applied to each sperm dataset to test the correlation between VAF and age. Comparison between the sperm age categories was subject to an initial Kruskal–Wallis test and for those with significant differences, and the Mann–Whitney *U* test was further used for categorical age comparison. The specific R-code used for these statistical tests is listed in Supplementary material online. Lastly, micropatterning data were subjected to one-way Analysis of Variance (ANOVA) testing to compare the means of the obtained contrast values.

### Testis Models

The models are based on what is known about human germline development and maturation. The models, and the parameter fitting and statistical testing strategies, reviewed below have all been previously published (Qin et al. 2007; Choi et al. 2008; Arnheim and Calabrese 2009; Choi et al. 2012; Shinde et al. 2013; Yoon et al. 2013; Arnheim and Calabrese 2016; Eboreime et al. 2022). The computer code to simulate the models is available at the following GitHub address: https://github.com/petercal/Germline-selection.

The symmetric hot spot model (Choi et al. 2012; Shinde et al. 2013; Yoon et al. 2013; Arnheim and Calabrese 2016; Eboreime et al. 2022) has 2 phases. The first phase, called the growth phase, models the testis from zygote formation to puberty. In this phase, there are 30 generations of symmetric divisions in which stem cells divide to form 2 stem cells. Since any new mutations are shared by daughter cells, due to the symmetric divisions a new mutation in the growth phase will form a mutation cluster (earlier mutations will form larger clusters). The next phase of the model, called the adult phase, models the testis postpuberty. In this phase, stem cells divide every 16 d (the age of the testis donor determines how many adult phase generations we simulate). Half of the stem cells divide symmetrically, and half of the stem cells differentiate (to form sperm precursors, which after a few subsequent divisions form sperm). The 50:50 split of symmetric divisions and differentiation ensures there is both a constant supply of testis stem cells and a constant production of sperm. A new mutation in the adult phase may produce a mutation cluster if the lineage where this mutation occurs subsequently experiences symmetric divisions, but this mutation cluster may also die out if many of these mutated stem cells then differentiate. This model has 1 free parameter: the mutation rate per cell division.

Separately for each mutation, we estimate the mutation rate per cell division that best matches the observed average testis mutation frequency. We do this by simulating the model at different mutation rates to find the value that maximizes the fraction of simulated average testis mutation frequencies that are within 5% of the observed average testis mutation frequency. We then simulate the model with this estimated mutation rate such that there are more than 100,000 simulations with the simulated average testis mutation frequencies within 5% of the observed average testis mutation frequency. For these simulations, we then compare the simulated maximum testis piece mutation frequency to the observed maximum testis piece mutation frequency. The *P*-value is the fraction of these simulations where the simulated maximum testis piece mutation frequency is greater than the observed maximum testis piece mutation frequency.

The symmetric selection model (Yoon et al. 2013; Arnheim and Calabrese 2016; Eboreime et al. 2022) also has 2 phases. The growth phase is identical to the hot spot model, and the adults phase is similar. The only difference is that in the adult phase of the selection model the mutated stem cells behave differently than the WT stem cells: the mutated stem cells (but not the WT stem cells) are more likely to divide symmetrically than to differentiate. This change makes the growth of mutation clusters more likely (and the dying out of mutation clusters less likely) than in the hot spot model. The bias between symmetric divisions and differentiation is the only free parameter in the model. In the selection model, we set the mutation rate per cell division to be equal to the rate that will produce the genome average mutation rate per generation in the case that there is no bias between symmetric divisions and differentiation (in this case the mutated and WT stem cells behave the same).

Similar to our strategy for the hot spot model, separately for each mutation, we estimate the bias term that best matches the observed average testis mutation frequency. We do this by simulating the model at different biases to find the value that maximizes the fraction of simulated average testis mutation frequencies that are within 5% of the observed average testis mutation frequency. We then simulate the model with this estimated bias such that there are more than 100,000 simulations with the simulated average testis mutation frequencies within 5% of the observed average testis mutation frequency. For these simulations, we then compare the simulated maximum testis piece mutation frequency to the observed maximum testis piece mutation frequency. The *P*-value is the fraction of these simulations where the simulated maximum testis piece mutation frequency is greater than the observed maximum testis piece mutation frequency.

## Supplementary Material

evae015_Supplementary_Data

## Data Availability

All the data produced in this study are available in Supplementary Material online.
