# Supplementary material for: Exploring FGFR3 Mutations in the Male Germline: Implications for Clonal Germline Expansions and Paternal Age-Related Dysplasias
Source: Genome Biol Evol. 2024 Feb 27;16(2):evae015. doi: 10.1093/gbe/evae015 (PMC10898338; doi:10.1093/gbe/evae015)
Supplement: evae015_Supplementary_Data [file evae015_supplementary_data.zip › SM Figures.pdf]

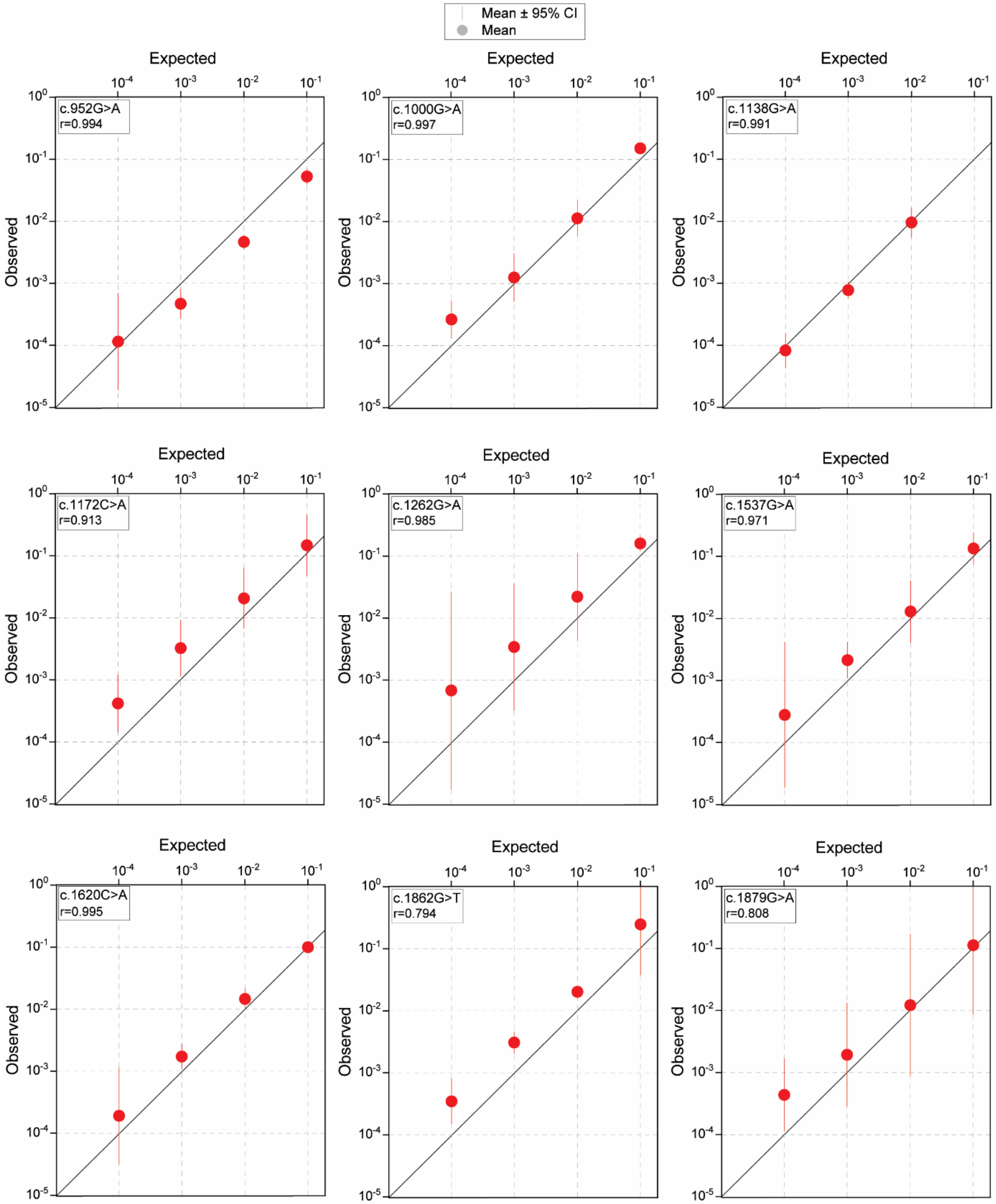

Supplementary Figure S1: Serial dilution experiments with positive controls for target variants using dPCR. Cell line encoding c.1620C>A and c.1138G>A or sequence-confirmed variant plasmids for the remaining target loci were spiked into wild-type human genomic DNA according to each dilution step. Pearson's R, *r*, was calculated to indicate the accuracy and reproducibility of both the in-house dPCR and commercial ddPCR.

A

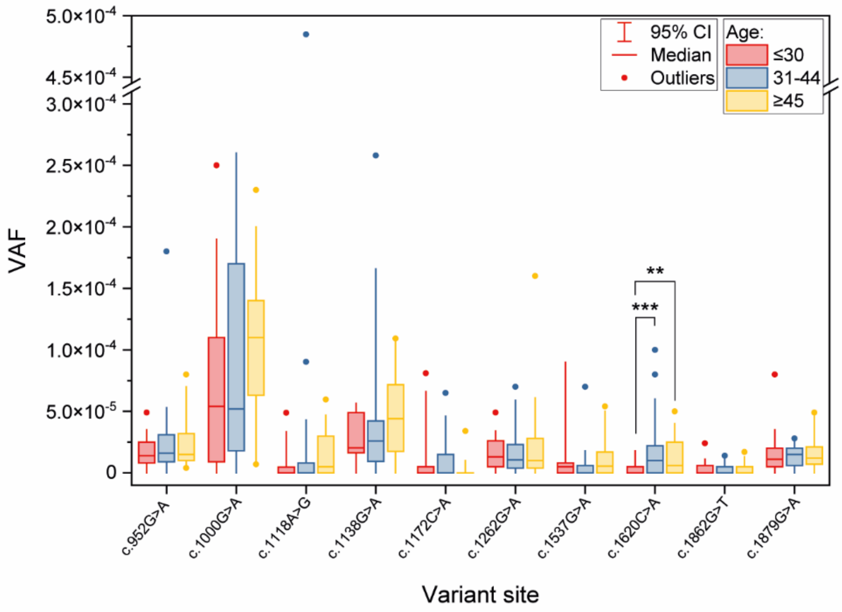

B

Variant locus:	n			Mean VAF			Min VAF			Max VAF		
**Age category:**	**≤30**	**31-44**	**≥45**	**≤30**	**31-44**	**≥45**	**≤30**	**31-44**	**≥45**	**≤30**	**31-44**	**≥45**
c.952G>A	26	40	26	1.6E-05	2.5E-05	2.5E-05	0	0	4.0E-06	4.9E-05	1.8E-04	8.0E-05
c.1000G>A	29	35	27	7.2E-05	9.4E-05	1.1E-04	0	0	7.0E-06	2.5E-04	2.6E-04	2.3E-04
c.1118A>G	33	42	23	5.5E-06	2.1E-05	1.4E-05	0	0	0	4.9E-05	4.8E-04	6.0E-05
c.1138G>A	10	26	20	2.7E-05	4.1E-05	4.7E-05	0	0	0	5.7E-05	2.6E-04	1.1E-04
c.1172C>A	25	30	24	1.1E-05	1.1E-05	2.7E-06	0	0	0	8.1E-05	6.5E-05	3E-05
c.1262G>A	30	38	31	1.5E-05	1.5E-05	2.0E-05	0	0	0	4.9E-05	7.0E-05	1.6E-04
c.1537G>A	33	31	28	1.1E-05	6.2E-06	1.1E-05	0	0	0	9.0E-05	7.0E-05	5.4E-05
c.1620C>A	32	47	29	3.7E-06	1.5E-05	1.3E-05	0	0	0	1.8E-05	1.0E-04	5.0E-05
c.1862G>T	30	36	27	2.7E-06	2.8E-06	2.5E-06	0	0	0	2.4E-05	1.4E-05	2E-05
c.1879G>A	25	34	20	1.5E-05	1.4E-05	1.5E-05	0	0	0	8.0E-05	2.8E-05	4.9E-05

Supplementary Figure S2: Variant allele frequency (VAF) of *FGFR3* variants in sperm donors of different age-categories. A. Mutation distribution in three age groups: younger (≤30), middle (31-44), and older (≥45) in sperm DNA for our candidate mutations. Tests for significant differences between the three age categories were performed with the Kruskal Wallis test; P-values were estimated using the Mann-Whitney-U test annotated as p≤0.01 (**) and p≤0.001 (***). **B.** Shown are sample sizes, mean and range (min and max) of each age-category.

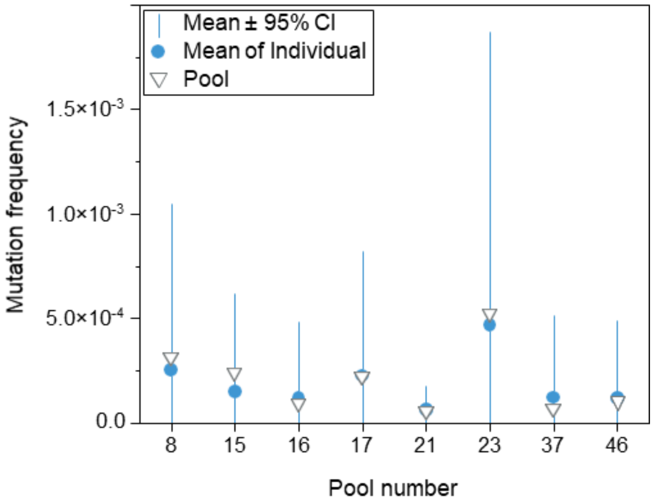

Supplementary Figure S3: Fine versus coarse pooling strategy. Mutation frequency accuracy test in eight different testis DNA pools for probe c.1620C>A (p.N540K) using two strategies: coarse vs fine pooling. Pool measurements (▼) represent the mutation frequency detected in the DNA of four testis pieces. The same testis pieces were measured individually and the respective mean (●) and 95% confidence intervals (CI) are represented. Data can be found in Supplementary Table S6.

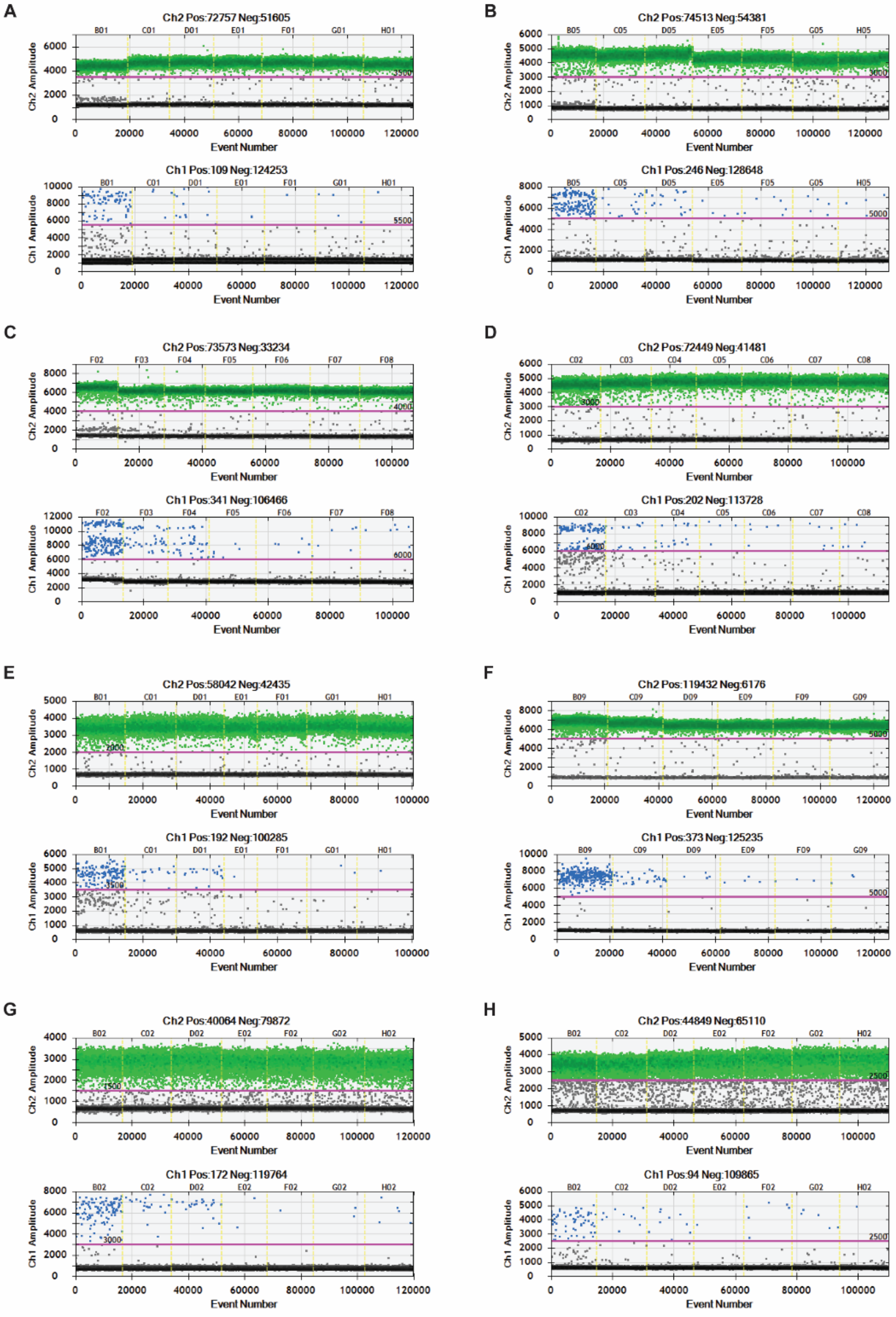

Supplementary Figure 4: Representation of QuantaSoft raw data used for ddPCR analysis for target variants. The data represents the mutant and wild-type droplets (channel 1 and channel 2, respectively) for the positive control samples of each of the target variants. Specifically, serial dilutions 1:100 are represented in well B, 1:1,000 in wells C and D, and 1:10,000 in wells E to H. The horizontal pink lines represent the set thresholds for variant calling for each of the target assays. Target variants detected with ddPCR: **A.** c.952G>A, **B.** c.1000G>A, **C.** c.1172C>A, **D.** c.1262G>A, **E.** c.1537G>A, **F.** c.1620C>A, **G.** c.1862G>T, and **H.** c.1879G>A. Event number corresponds to the number of droplets detected in each assay. Ch1: Channel 1. Ch2: Channel 2.
