# Supplementary material for: Exploring FGFR3 Mutations in the Male Germline: Implications for Clonal Germline Expansions and Paternal Age-Related Dysplasias
Source: Genome Biol Evol. 2024 Feb 27;16(2):evae015. doi: 10.1093/gbe/evae015 (PMC10898338; doi:10.1093/gbe/evae015)
Supplement: evae015_Supplementary_Data [file evae015_supplementary_data.zip › Supplementary_Methods_revised.docx]

# Supplementary Methods

## WT DNA Plasmid

In brief, a *FGFR3* region of 1887bp was amplified from 10ng of human genomic DNA in a 50µl reaction containing 0.5µM of each primer (F-ACH-88bp and R-TDII_BA; see Table S11), 1x Phusion HF Buffer (ThermoFisher Scientific), 0.1U Phusion Hot Start II High-Fidelity DNA polymerase (ThermoFisher Scientific), and 0.2mM of dNTPs. The reaction was carried out with an initial heating step of 98°C for 1 minute, followed by 40 cycles at 98°C for 15 seconds, 68°C for 15 seconds, and 72°C for 30 seconds. The 3’ A-overhangs were added with 1U of One Taq in a 15µl reaction and further incubated at 72°C for 10 minutes. The purified amplicon was cloned into a pCR2.1 vector using the TA-cloning Kit (Invitrogen) and transformed into XL1-blue competent cells. The plasmid was extracted with a standard plasmid extraction protocol detailed in (Arbeithuber, et al. 2016). In short, cells were pelleted, resuspended and lysed with an alkaline, detergent solution and the DNA was obtained by acetate/ethanol precipitation.

## *E.coli* DNA.

XL1 *E. coli* blue cells were grown in 15ml LB-medium overnight at 37°C until reaching an OD600 of two. A 3ml cell suspension was then centrifuged at 8000g for 30 sec and the cells were resuspended in the 10% leftover supernatant before adding 600µl cell lysis solution (Gentra Puregene Cell Kit), 24µl 1M DTT, and 2µl proteinase K (QIAGEN 20mg/ml) and incubated overnight at 37°C. RNase treatment was performed by adding 3µl of a 4mg/ml RNase A solution and incubated for 15 minutes at 37°C. The reaction was put on ice for ~15 minutes and 200 µl of protein precipitation solution was added snipping the tube vigorously for 1 minute, followed by two consecutive centrifugation steps at 13,000g for 20 min. The DNA was pelleted by adding 600µl of isopropanol and 1 µL of glycogen solution to the supernatant. At this step a visible DNA bundle was formed when mixing gently. Then, the reaction was centrifuged for 30min at 13,000g, and the pellet was washed with 600µl 70% ethanol and left to dry for 3 minutes. The DNA was resuspended in TE 7.5 (50µl) overnight.

**Site-Direct Mutagenesis (SDM)**

To introduce site-specific single-point mutations in our positive controls, a plasmid containing a genomic fragment (pCR2.1 vector) or only the coding sequence (pcDNA3.1; mGFP-FGFR3 expression vector (Hartl et al., 2023)) of the *FGFR3* gene was amplified using the high-fidelity polymerase Phusion HS II (2U/µl; ThermoFisher Scientific, #F-549L). Oligonucleotide pairs specifically designed (back-to-back) for SDM (see Table S9), carried a 5’-phosphate modification in one of the primers and were used to introduce a site-directed base change that created a non-synonymous target mutation in the *FGFR3* gene. Note that for the coding sequence (CDS; pcDNA3.1) plasmid, a silent mutation was added in close proximity to the target mutation to detect potential aerosol contaminations of these plasmids in ultra-sensitive sequencing technologies used in our laboratory. To avoid potential downstream consequences due to different codon usage, we chose codons for the silent mutations with a similar usage frequency in humans.

Amplification for site-directed mutagenesis of the mGFP-FGFR3 expression plasmid was carried out on mGFP-5xGGS-FGFR3_pcDNA3.1/Hygro(+) expression vector. The entire 8724 bp vector, containing as insert a strong Kozak sequence (6 bp), the FGFR3 signal peptide sequence (66 bp), the mGFP coding sequence (714 bp), a 5x GGS linker (45 bp) followed by the FGFR3 extracellular, transmembrane and intracellular domain (2358 bp), was amplified using back-to-back primers (see Table S9 for detailed sequences) to insert the desired mutations. 5ng of the template vector was added to the PCR as a circular plasmid. 0.3U/µl of Phusion HS II High-Fidelity DNA Polymerase (ThermoScientific) were used in a 25µl reaction in 1x HF-reaction buffer supplemented with 0.3%DMSO, 0.2mM dNTPs (Biozym) and 0.4µM of each primer. The PCR started with an initial heating step of 98°C for 2 minutes, followed by 25 cycles at 98°C for 10 seconds, an annealing step at 63.9/64°C for 15 seconds, and 72°C for 4.5 minutes, concluded by a final elongation step of 3 minutes at 72°C in a T100 Thermal Cycler (BioRad). The correct length of the amplicon(s) was assessed via gel electrophoresis. See Table S9 for site-specific annealing temperatures.

Amplification for site-directed mutagenesis of the *FGFR3* genomic plasmid was done in genomic DNA plasmid (pCR2.1 vector; see above). The entire 5817 bp vector was amplified using back-to-back primers (see Supplementary Table S10) to insert the desired mutations. PCR was performed as mentioned for the expression plasmid, except the elongation time was only 3 min in each cycle.

Next, PCR products forming partially nicked circular DNA were ligated for which 5µl of the PCRs were used directly (without purification steps) in a 20µl reaction in 1x T4 DNA Ligase Reaction Buffer (NEB) with 400U T4 DNA Ligase (NEB). The reaction was mixed by pipetting and incubated first for 15 minutes at room temperature (RT), followed by incubation at 16°C for up to 2 hours, and finally, placed on ice for at least 10 minutes.

DpnI digestion was as follows: 10µl of the ligation reaction were filled up to 20µl with nuclease-free water and supplemented with 1x CutSmart buffer and 10U of DpnI (NEB) and the methylated template plasmid was digested using an incubation time of 1 hour at 37°C. Next, the 20µl of the ligation reaction was transformed into 50µl of chemically competent *E. coli* NEB 10-beta (NEB) according to the manufacturer’s instructions and plated on LB-agar containing 100µg/mL of ampicillin. Ampicillin-resistant clones were screened by colony PCR for the formation of a correctly sized fragment containing the mutated site.

Different primer sets were used (see table below) for colony PCR screening, depending on the location of the mutated site. OneTaq-HS® polymerase (0.025U/µl; NEB) was used in a 20µl reaction in 1x standard buffer supplemented with 0.2mM dNTPs (Biozym) and 0.5µM of each primer. As a template, the single colonies were dipped into the PCR master mix. The PCR started with an initial heating step of 94°C for 5 minutes, followed by 30 cycles at 94°C for 15 seconds, annealing at varying temperatures given by the primer combinations for 15 seconds, and 68°C for varying amounts of times given by the length of the intended product, concluded by a final elongation step of 5 minutes at 68°C. The correct length of the amplicon was assessed via gel electrophoresis and the positive clones were inoculated in 5mL LB medium containing 100µg/mL ampicillin to grow in an overnight culture, shaking at 37°C. 4 mL of the culture were harvested by centrifugation and a plasmid Miniprep was performed using the PureYield Plasmid Miniprep System (Promega) according to manufacturer’s instructions. The pure plasmids were sent to sequencing at LGC genomics.

## DNA lesions and negative controls

Unfortunately, there is no appropriate mutant-free DNA that can be used as a negative control to accurately establish the sensitivity of ddPCR and the level of technical noise at each site and sequence context analyzed here. *E. coli* has a high-fidelity replication machinery with an error rate as low as 5.4x10^-10^ per base pair per replication (Drake et al., 1998). In theory, this low error replication rate would make sequence-confirmed wild-type plasmids a hypothetically mutation-free control. In our measurements, this was not always the case with oxidation and heat exposure possibly contributing to the number of mutations to levels of 10^-6^ to 10^-5^ from these common DNA lesions (Arbeithuber et al., 2016; Jee et al., 2016).

We hypothesize that mutation counts observed in the sequence confirmed wild-type plasmids is a result of DNA lesions introduced during freeze-thawing cycles (e.g., either during the DNA extraction) (Arbeithuber et al., 2016; Lindahl and Nyberg, 1974; Ohsako et al., 1997; Ross et al., 1990). Note also that most of our variants are missense substitutions at CpG sites, which can carry a high number of DNA lesions as a result of cytosine deamination and result in strong to weak transition mutations (C>T or G>A). Further, we used a different DNA extraction protocol for this WT plasmid than for the sperm or testis samples that could introduce more lesions.

It is possible to use repair enzymes like uracil base-excision enzymes (e.g., uracil DNA glycosylase (UNG)) to reduce the number of artefacts caused by cytosine deamination resulting in uracil (Hofreiter et al., 2001; Lindahl, 1982; Lindahl et al., 1977; Walsh and Xu, 2006; Zharkov et al., 2010). But this enzyme is not useful, if the CpG sites are methylated (5-methylcytosine) as is often the case in sperm (Cooper et al., 2010; Jenkins et al., 2014; Nilsson et al., 2018).

In order to examine the possible contribution of false positives in our sperm and testis samples, we merged the WT counts of individual testis pieces or sperm donors without mutations for each variant site. The contribution of false positives should be similar among measurements since all samples had the same treatment. The number of WT genomes in samples without any mutant counts ranged from ~3x10^6^ to 10^7^ depending on the variant. From these numbers, we can conclude that the contribution of lesions at VAF levels of 10^-5^ is negligible (see Supplementary Table S3).

## Data analysis for the micropatterning experiments

Images of patterned cells were analyzed as described in (Hartl et al., 2023). In short, the mGFP-FGFR3 enriched and immobilized to the micropatterns, represent (I_ON,mGFP_), leaving other regions depleted of mGFP-FGFR3 (I_OFF,mGFP_). The co-recruitment of GRB2-mScarlet to mGFP-FGFR3 patterns was imaged using total internal reflection fluorescence (TIRF) microscopy. “ON” and “OFF” selection masks were determined by comparing the images recorded in green and red channel representing the FGFR3-mGFP and GRB2-mScarlet pattern, respectively. All “ON” and all “OFF” areas of one cell (usually 4-9) were pooled and the mean pixel intensity values of “ON” and “OFF” regions, I_ON_ and I_OFF_, were used for further analysis. The contrast value was then determined separately for each cell and color channel with the fluorescence contrast, $C= \frac{I_{ON}- I_{OFF}}{I_{ON}- bg}$ serving as a measure for the activation. Note that the background intensity (bg) was assessed from a recorded image without any illumination.

For each experiment, we collected at least 30 individual data points (cells) from at least 3 independent measurements (transfections) that were merged. Median and average contrast values with exact cell numbers are listed in Supplementary Table S8.

## In-house dPCR (BEA: bead-emulsion amplification)-

## This in-house amplification followed protocols published previously and described in detail in (Striedner, et al. 2024). In brief, testis, sperm, or plasmid DNA samples underwent digestion with CviQI (NEB) to generate approximately 500-base pair fragments containing the target sites. The emulsion was prepared using the Dow-Corning components as described in (Palzenberger, et al. 2017). The beads were mixed with the aqueous PCR phase and the oil phase followed by PCR. Beads were washed, labelled, and immobilized on a microscope slide for scanning. Only ~10% of beads amplifying a product were considered in each experiment to ensure accurate mutation number estimation: After the initial scan, probes were washed off, and beads were re-labelled with probes of interest using a hybridization chamber and in situ PCR block.

Variant c.1118: After scanning the beads for two different loci as detailed in (Striedner, et al. 2024), the same beads were washed and re-scanned for variant c.1118A>G. The probes were washed off a third time and re-labelled with the probes c.1118A wt A647 (/5Alex647N/GACGAGGCGGGCAGTGT*G*T*A), c.1118A>G mut A532 (/5Alex532N/ACGAGGCGGGCAGTGT*G*T*G). Labelling occurred in a chamber kept at 95°C for 2 minutes followed by 63°C for 5 minutes, 72°C for 5 minutes and kept at 75°C until unextended probes were washed off. Note that the asterisks denote phosphorothioate bonds. For the dye switch, the probes were washed off and the beads were re-labelled with dye-switch probes: c.1118A wt A532 (/5Alex532N/GA CGAGGC GGGCAGTGT*G*T*A), c.1118A>G mut A647 (/5Alex532N/ACGAGGCG GGCAGTGT*G*T*G). Since the beads were immobilized on an acrylamide array, it was possible to re-scan the beads without losing the positional information of each bead.

For each experiment two sets with 3 images each were analysed for both the normal scan and the dye switch scan. Brightfield images between scans were aligned by computing the normalized cross correlation matrix of pairs of images as described previously (Boulanger, et al. 2012). Using a combined masked constructed from the combination of both bright fields was employed to extract the intensity values for defined regions of interests (ROI) representing each bead across the fluorescent channels (two per scan). The average pixel intensities for each ROI was compiled for each fluorescent channel to classify the beads into different clusters (10, 01, 11, 00) based on the signal intensity for each fluorophore (the 11 cluster represent beads fluorescing in both channels, and the 00 cluster represent empty beads with no fluorescence). The classification scheme was based on a Gaussian Mixture model and normalization parameters described in detail previously (Boulanger, et al. 2012). Wild type beads were identified as beads classified as 1001 and mutants as 0110.

## BEA: Statistical analysis.

Spearman’s correlation coefficient was used to test the correlation between variant allele frequency (VAF) and sperm donors’ age. Kruskal-Wallis was used to test VAF differences between the three age categories in sperm donors. Please note that all VAF presented in this work were Poisson corrected according to the following formula:

$$\lambda=-\ln\begin{aligned} (1-(\frac{Mutant}{Mutant+Wild-type}) \end{aligned}$$

**Statistical analysis**

We used the following R-code for the statistical tests:

Mann-Whitney-U:

wilcox.test(data$mut.freq~data$diagnosis)$p.val

Kruskal-Wallis:

kruskal.test(data$mio.ml~data$diagnosis)$p.val

Spearman's test:

cor.test(data$mut.freq, data$mio.ml, method = "spearman")$p.val

cor.test(data$mut.freq, data$Age, method = c("spearman"))

## References

Arbeithuber B, Makova KD, Tiemann-Boege I. 2016. Artifactual mutations resulting from DNA lesions limit detection levels in ultrasensitive sequencing applications. DNA Res 23:547-559.

Boulanger J, Muresan L, Tiemann-Boege I. 2012. Massively parallel haplotyping on microscopic beads for the high-throughput phase analysis of single molecules. PLoS One 7:e36064.

Palzenberger E, Reinhardt R, Muresan L, Palaoro B, Tiemann-Boege I. 2017. Discovery of Rare Haplotypes by Typing Millions of Single-Molecules with Bead Emulsion Haplotyping (BEH). Methods Mol Biol 1551:273-305.

Striedner Y, Arbeithuber B, Moura S, Nowak E, Reinhardt R, Muresan L, Salazar R, Ebner T, Tiemann-Boege I. 2024. Exploring the Micro-Mosaic Landscape of FGFR3 Mutations in the Ageing Male Germline and Its Implications in Meiotic Differentiation. Preprint.
